# Multi-Omics Approaches and Radiation on Lipid Metabolism in Toothed Whales

**DOI:** 10.3390/life11040364

**Published:** 2021-04-20

**Authors:** Jayan D. M. Senevirathna, Shuichi Asakawa

**Affiliations:** 1 Laboratory of Aquatic Molecular Biology and Biotechnology, Department of Aquatic Bioscience, Graduate School of Agricultural and Life Sciences, The University of Tokyo, Tokyo 113-8657, Japan; asakawa@g.ecc.u-tokyo.ac.jp; 2 Department of Animal Science, Faculty of Animal Science and Export Agriculture, Uva Wellassa University, Badulla 90000, Sri Lanka

**Keywords:** genomics, transcriptomics, proteomics, lipidomics, radiation, cetaceans

## Abstract

Lipid synthesis pathways of toothed whales have evolved since their movement from the terrestrial to marine environment. The synthesis and function of these endogenous lipids and affecting factors are still little understood. In this review, we focused on different omics approaches and techniques to investigate lipid metabolism and radiation impacts on lipids in toothed whales. The selected literature was screened, and capacities, possibilities, and future approaches for identifying unusual lipid synthesis pathways by omics were evaluated. Omics approaches were categorized into the four major disciplines: lipidomics, transcriptomics, genomics, and proteomics. Genomics and transcriptomics can together identify genes related to unique lipid synthesis. As lipids interact with proteins in the animal body, lipidomics, and proteomics can correlate by creating lipid-binding proteome maps to elucidate metabolism pathways. In lipidomics studies, recent mass spectroscopic methods can address lipid profiles; however, the determination of structures of lipids are challenging. As an environmental stress, the acoustic radiation has a significant effect on the alteration of lipid profiles. Radiation studies in different omics approaches revealed the necessity of multi-omics applications. This review concluded that a combination of many of the omics areas may elucidate the metabolism of lipids and possible hazards on lipids in toothed whales by radiation.

## 1. Introduction

Marine mammals are megafauna that live in the deep sea and coastal environments after leaving the land between 50 and 25 million years ago; the modern whale fully adapted to the aquatic life around 10 million years ago [1]. According to their body features, feeding habits, and other factors, they are divided into three major orders; namely, Pinnipidia (seals, sea lions, walruses), Cetacea (whales, dolphins, and porpoises), and Sirenia (manatee and dugong) [2]. With this transition, they have developed numerous adaptations to survive in the ocean. A significant feature of these animals is the presence of a fat layer around the body, called the blubber. This blubber of marine mammals consists of subcutaneous adipose tissue and connective tissue that has three major functions: to store energy, to control buoyancy, and body heat regulation [3]. Moreover, a study has suggested that the brown adipose tissue of blubber and its protein, uncoupling protein 1, have a significant role in functioning like an insulation blanket in the cetacean body [4]. Toothed whales are a group in the order cetacea that includes some whales, and all dolphins and porpoises. They have special adaptations for the marine environment, such as echolocation for foraging and communication. Toothed whales have unique acoustic fats in the head region, namely, melon and jaw/mandibular fats, and it has been documented that those fats are involved in echolocation [5,6,7,8,9]. The composition and formation of these acoustic fats in toothed whales have differences with the blubber in the same animal. These specialized fats may be involved in the metabolism for various adaptations to their aquatic life. However, the origin of these specialized acoustic adipose tissue and the composition of these fats is still debated with limited scientific facts; therefore, comprehensive multi-scale approaches are needed to elucidate this kind of biological phenomena.

Lipids are a source of energy, commonly stored in specialized cells called adipocytes, as a form of triacylglycerol (TAG), in vertebrates [10]. TAG and wax esters (WE) are two common lipids usually stored in toothed whales’ acoustic adipose tissues. The storing of wax esters in these animals is unusual compared to other mammals. Examples of naturally occurring wax esters are found in plant epicuticles, bees’ wax, and the spermaceti oil of toothed whales. Waxes of toothed whales consist of uncommon fatty acids (FA) and fatty alcohols (FAlc), which contain special features that can be used in various industries. However, the synthesis of these lipids is currently not well understood, and the number of studies has been limited [11]. Usually, lipids are stored in adipocytes by various processes like lipoprotein hydrolysis, fatty acid uptake, *de novo* synthesis, and etherification [12]. The composition of TAG and WE in toothed whale species varies; therefore, the biological synthesis of these lipids could occur via different pathways [8]. In addition, the mammalian adipose tissue consists of many kinds of cells, collectively called adipokines, and is a complex tissue for the study of metabolism [13]. Therefore, more studies on understanding the uniformity, physical properties of lipid distribution in toothed whales, complete ontogenetic series, and metabolism of these fats have been recommended [8,14]. With this complexity of lipid species in toothed whales, a comprehensive study of the metabolism of these lipids will be highly interesting; however, it has not been tested yet.

The lipidome of the cell or cell compartment of an organism is an indicator of synthetic pathways of the organism and its environment [15]. There are inborn mechanisms in any organism to react to environmental stresses at a molecular level, such as fatty acids, where scientists can observe, measure, analyze and predict. These reactions are also important in understanding human and environmental interactions, and for predicting environmental changes, climate change, environmental toxicology, biotechnology and bioengineering, and many other aspects. Several factors, such as genes, environmental factors, and gene and environment interactions, can also affect the synthesis of the endogenous lipid profiles in mammals. Therefore, the lipid profile of toothed whales can be changed with dietary lipids, *de novo* lipogenesis, enzymes, desaturation, physical forces, and the incorporation of fatty acids into more complex lipid molecules [11,16,17]. In addition, changes in lipid metabolism in animals can occur due to anthropogenic or natural factors like radiation [18].

Radiation is the releasing of energy as waves or particles in any environment, that can cause biological impacts in any form of life. There are two main types of radiations, including ionizing and non-ionizing (extremely low frequency and radiofrequency) [19]. Sources of radiation are man-made (industries, power plants, telecommunication, and sonar) or natural (solar, space, and cosmic). A study has found that ultraviolet radiation affects cutaneous lipids and bioactive lipids for skin inflammation in humans [20]. Several types of anthropogenic radiation and radioactivities can impact marine life, including cetaceans. Moreover, different types of radiation and radioactivity underwater are environmental factors that can alter the specialized lipids of toothed whales. The ionizing radiation can also impact biological materials on cellular levels and organ levels, and have other low-level effects, such as cancers [21]. Although we could find direct evidence for the biological impacts of ionizing radiation in the literature, it is possible that ionizing radiation can also impact lipid metabolism in toothed whales. Particularly, a novel study puts forward that ionizing radiation induces reactive oxygen species and ACSL4 expression, a lipid metabolism enzyme resulting in elevated lipid peroxidation and ferroptosis in cancer cell-lines [22].

This review is mainly focused on acoustic radiation as an environmental factor that can influence lipids in toothed whales. Especially in toothed whales, this type of radiation may interrupt echolocation behavior while damaging special acoustic fats in the head region. For example, non-ionizing radiation, such as acoustic pollution, can impact various organs in whales’ bodies and diminish the reproduction of these marine mammals [23]. The effects of noise pollution in various gene functions from the hypothalamic–pituitary–adrenal and hypothalamic–pituitary–gonadal (HPA) axes in animal bodies have been largely discussed; however, more studies are needed on toothed whales. Sonar and its underwater acoustic pollution is a highly concerning environmental stressor. The acoustic radiation of sonar can damage melon, and other lipid tissues in toothed whales and cause hearing loss [24]. Moreover, they have suggested that this anthropogenic radiation may also damage body tissues, effect behavioral changes, damage the dive cycle, damage breathing patterns, damage the sound production rate, and have negative effects on the energy budget. These changes are very complex to study at the molecular level and hard to understand metabolically, hence, the integration of several fields of studies like physical, chemical, and biological in the micro-environment is recommended. Experimental research on a model animal (female and male infants of rabbits) has been conducted to identify the effects of radiofrequency (RF) radiation on DNA and lipid damage [25]. Finally, they have identified that the global system for mobile telecommunication (GSM-like) RF radiation can cause attacking free radicals to DNA, and lipids, ultimately changing their biochemical structures [25]. Accordingly, we also suggest that radiation can act as a highly potential environmental stressor on lipid metabolism in other animals. Therefore, we should pay more attention to the research on the effects of radiation on lipids of toothed whales to identify its biological impacts in the polluted marine environment.

Several factors that can produce big data need to be concerned in studies of animal lipid metabolism; hence, we recommend that the integration of various kinds of molecular data is suitable and more accurate in studies on complex lipid metabolism in toothed whales and its changes due to environmental stressors. A comprehensive understanding of the lipid metabolism of these animals requires identification of molecular changes in different levels, such as genomics, transcriptomics, proteomics, and metabolomics (lipidomics), commonly called “multi-omics”. In this approach, a variety of data generated from different omics tools are concerned for analysis and predictions [26]. Toothed whales have a rich diversity, including around 70 species, and they live mainly in different habitats of the marine environment; some are cosmopolitans, migratory, different sizes, and dive into various depths (sperm whale is the deepest diver). Single omics approaches can generate data comparative to many factors, according to the interest of the research design; then, integration of data in the multi-omics approach gives an overview of the experimental results as a whole for better interpretation. Moreover, these omics data are useful to understand marine mammals’ health, and their environmental health [27].

In the past decades, lipidomics studies have been conducted to uncover the different aspects of lipid diversity, composition, evolution, and metabolism in toothed whales. Integrated multi-omics approaches could be tested to understand the complete set of genetics, transcriptomics, and to uncover the lipid species [28]. A recent study has identified abnormal lipid metabolisms, such as suppression of fatty acid metabolism, promotion and metabolism of glycolipids and phospholipids, using multi-omics analysis [29]. However, detailed omics projects on cetaceans on metabolism are limited due to constraints on sampling fresh tissues. Due to the limited number of combined omics data on toothed whales for functional enrichments, we had to rely on the data, methods, and technologies tested for other species. The advantage of multi-omics’ is the various types of big data that can be integrated to collect a variety of biological information, unrevealing hidden mechanisms in lives. A wide variety of bioinformatics, including mathematical arrays, algorithms, statistics, computational skills, programming, artificial intelligence (AI), deep learning (DL), and machine learning (ML) techniques, are currently adapting to analyze and integrate omics data into a single platform for understanding metabolic pathways and diseases in various animals, as well as humans. Mostly these techniques were used for the identification of cetacean in the wild [30,31]. Recently, the ML technique has been applied in the detection and classification of sperm whales using bioacoustics [32]. Another study has identified the impact of polychlorinated biphenyls (PCBs) on the transcriptome of the common bottlenose dolphin [33]. Therefore, it is evident that the ML has a potential to identify lipid profile changes in toothed whales due to various factors. In addition, novel omics technologies can be applied to understand changes of molecules, from DNA to protein and their modifications, because the effects of radiation on biological systems can clearly be understood [34]. Another important aspect of multi-omics is a deeper understanding of processes, dynamic interactions, molecular frameworks, novel molecular mechanisms, novel pathways, biomarkers, and drug targets. Multi-omics can identify the activation and inactivation of enzymes in adipocytes, and ML can link the affecting factors for these biomolecular changes. Therefore, the biomolecular damages caused by radiation on toothed whales can be analyzed by applying the combined approach of multi-omics and ML in the future.

## 2. Methodology

**Figure 1:**
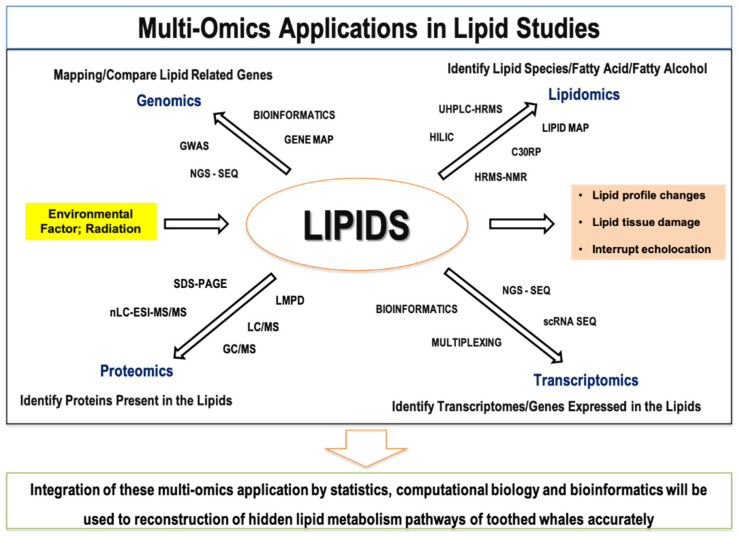
Integrated multi-omics approach for identification of lipid metabolic pathways. Note; GWAS (Genome-wide association study), SDS-PAGE (sodium dodecyl sulphate-polyacrylamide gel electrophoresis), GC/MS (gas chromatography/mass spectrometry), LC/MS (liquid chromatography/mass spectrometry), UHPLC-HRMS (ultra-high-performance liquid chromatography-high-resolution mass spectrometry), HILIC (hydrophilic interaction liquid chromatography), C30RP (C30 reversed-phase chromatography), HRMS-NMR (high-resolution mass spectrometry-nuclear magnetic resonance spectroscopy), LMPD (LIPID MAPS proteome database), LC/ESI-MS/MS (liquid chromatography electrospray ionization tandem mass spectrometric), scRNA-seq (single-cell RNA sequencing), NGS-SEQ (next generation sequencing).

In this review, we focused on identifying technologies in lipid-related genomics, transcriptomics, proteomics, lipidomics, and radiation studies that could elucidate the relationships, gaps, and opportunities for further studies on lipid metabolism in toothed whales. We searched articles in Google Scholar, and PubMed databases using various combinations of keywords, such as “-omics”, “lipids”, “fats”, “adipose tissue”, “toothed whales”, “lipid metabolism”, “acoustic radiation”, and “radiation”, and around 150 references were selected based on relatedness to lipid metabolism of toothed whales to discuss in this review. During the literature survey, we identified current approaches to study lipid metabolism, research gaps, limitations, and future aspects. Based on the information gathered, we propose a multi-omics approach by integrating omics technologies to study the unique lipid synthesis pathways and the possible impacts of radiation on the lipid metabolism of toothed whales and achieve further understanding (Figure 1). Moreover, future focuses in different omics studies were summarized to understand the applicability of multi-omics applications on lipid metabolism in the future (Supplementary Table S1).

## 3. Results and Discussion

Lipid patterns in toothed whales have been studied for many species during the past decades. A study found high levels of isovaleric lipids in the families Delphinidae, Phocoenidae, and Monodontidae while long-chain lipids were found in Ziphiidae, Physeteridae, and Platanistidae [7]. Later, the distribution of unusual branched-chain and wax ester lipids was identified in the mandibular fats of bottlenose dolphins [35]. Recently, Koopman (2018) has suggested that the importance of identifying specific molecular mechanisms in the synthesis of wax esters and branched-chained fatty acids in toothed whales [11]. It is also evident that some enzymes known to participate in wax ester biosynthesis have been showed pseudogenization in cetaceans [36]. As indicated in Figure 1, multi-omics is a combined approach of high-throughput technologies in genomics, transcriptomics, proteomics, and metabolomics [37]. Due to the limitations of findings from fresh samples of toothed whales, the design of this kind of study is critical. However, sample collections from strandings, fisheries, and biopsy collections are possible ways to study the toothed whales’ metabolism. Therefore, a single tissue sample can be used for several omics analyses, such as isolating DNA for genome analysis, isolating RNAs for making tissue-specific cDNA libraries and de novo transcriptomics analysis, protein extractions for identifying enzymes involved in lipid metabolism, and extracting TAG, and wax esters to elucidate lipidomics. The big data produced in these omics’ platforms can be integrated by bioinformatics tools and, also by applying DL, and ML techniques with the support of other AIs. Moreover, lipid profile changes, and biomolecular damages of stranding toothed whales can be subjected to comparative analyses to identify the possible impacts of acoustic radiation and further developments. Based on the present knowledge, and technology, factors affecting lipid profile changes in toothed whales can be predicted, and the unique lipid metabolism pathways of these animals will be revealed in the future.

### 3.1. Omics Applications on Lipid Metabolism and Radiation

#### 3.1.1. Genomics

Genomics is one of the fundamental omics approaches. It is the science of studying genomes (DNA sequences) and nucleotide variants in their coding, and non-coding regions that provide central information of the metabolism of an organism. It has expanded to more functional levels to study gene expression, and gene interactions with proteins [38]. Toothed whales show convergent evolutionary adaptations. A study has highlighted that the parallel molecular changes in coding genes cause phenotypic changes of the animal that are favorable for aquatic life [39]. Interestingly they have identified that MYH7B, S100A9, and GPR97 genes were specific to toothed whales and not present in baleen whales. The evolutionary context of lipid metabolisms in cetaceans has been described, and 144 genes have been identified in the lipid metabolism of cetartiodactyl (cetaceans and artiodactyls), including toothed whales by genome comparison [40]. The triacylglycerol metabolism of cetaceans has been investigated using 88 related genes, and 41 were identified as being involved with triacylglycerol synthesis and lipolysis processes [41]. The importance of genes of cetacean species were described for aquatic adaptations. Several studies have been carried out to elucidate the gene loss in marine mammals in transforming to the aquatic environment, including the loss of the adenosine monophosphate deaminase gene family (AMPD3), a change that may have improved oxygen transport in sperm whales [42]. A study on bottlenose dolphins has identified significantly enriched genes for lipid transportation and localization [43]. The gene leptin (Lep) is expressed differently in seasonal and age-related variations in lipolysis in bowhead and beluga whales [3]. Additionally, the results of studies on humpback whale adaptations have been considered in human cancer therapy research [44].

Genes for lipid metabolism and de novo synthesis have been identified in many organisms. The FadR gene in *Escherichia coli* is a transcriptional regulator of fatty acid biosynthesis [45]. Expression of the WSD1 gene in *E. coli* and *Saccharomyces cerevisiae* contributes to the production of wax esters, indicating that WSD1 mainly functions as a wax synthase [46]. The expression of three genes is important in lipid metabolisms, such as lipoprotein lipase (LPL), muscle carnitine palmitoyl transferase-1 (mCPT1), and fatty acid-binding protein (FABP), as they correlate with the expression of PPAR-gamma in muscle samples of human [24]. The mesoderm-specific transcript (Mest) is identified in mice as a lipase; high expression of Mest leads to excess lipase activity, and lipid accumulation. If Mest occurs in non-lipid tissue, it can cause lipotoxicity and could be a subject for study in de novo lipid synthesis [47]. Genetic screens of *Caenorhabditis elegans* show that the fat regulation is performed by a complex gene network with some other functions, and that the fat regulatory pathways are similar to those in mammals [48]. 

Next-generation sequencing (NGS) provides new insight for genomics analyses to uncover genomes in de novo. In a detailed analysis of genomics, it is advisable to use more than one genome as a reference to get effective results of gene expression and function [49]. Therefore, reference genomes have been created for many species, including toothed whales with current advancements of short-read and long-read sequencing techniques. Genome-wide association studies (GWAS) are used in comparative genomics to identify potential genes for lipid synthesis. Several lipid metabolism related genes, such as LDLRAP1, APOA5, ANGPLT3/4, and PCSK9, are currently being revealed by GWAS. In the unsolved main lipid pathways, these adaptor proteins were discovered, and GWAS have shown many new loci [50]. However, monogenic approaches for identifying complex lipid traits are not suitable because of the polygenic origin of these complex traits. Therefore, the combination of large-scale data and sophisticated computer analyses using novel bioinformatics techniques are needed to identify candidate genes in lipid metabolism [50]. The identification of interaction of genomics and phenomics is an approach for understanding different lipid species. Trans-omics (reconstruction of global biochemical network with multi-omics) analyses have been used to identify PSMD9 as a previously unknown lipid regulator [51]. In contrast, this information shows the importance of genomics and combinations with other layers to future studies of genomics on lipids in toothed whales.

For many years, the genotoxic effects of radiation have been discussed. Its impacts on changing nucleic acid chemistry and biological consequences are also identified in different levels [34,52,53] and sometimes called radiogenomics [54,55,56]. Rare genetic diseases have been recorded due to radiation, such as ataxia-telangiectasia, Nijmegen breakage syndrome, ataxia telangiectasia-like disorder and DNA ligase IV deficiency in humans [57]. In another study, the effect of radiation in the gene expression of IL4I1, SERPINE1, TP53, RELA, and CDKN1A and their effect on the NF-kB pathway have been revealed [58,59,60]. A study has identified RUNX1 (a regulator of the NF-kB pathway), which plays a critical role in lipopolysaccharide-induced lung inflammation [61]. The mechanism of action of RF radiation on DNA/protein expression (heat–shock protein) has been largely discussed [62]. The radical-pair photoreceptor hypothesis is another mechanism that explains possible ways of radiation can inhibit DNA synthesis and transcription and finally, cause carcinogenesis [25]. However, we did not find any direct references related to the effects of any radiation on lipid metabolic genes in toothed whales, and hence, future investigations are needed in this aspect. Genome level mutations and alterations can happen by radiation, such as in the form of micro-waves; therefore, research on the physio-chemical changes of DNA should be conducted in the future. Genomics research on radiation should be implemented by applying multi-scale new approaches.

#### 3.1.2. Transcriptomics

Currently, transcriptomics (the study of RNAs) has the potential to study various types of RNA expressed at the cellular level. The transcriptomics analysis of whales is limited due to difficulties in finding fresh tissue samples. No cDNA library from fats or liver is currently available for any cetacean species to facilitate the identification of up-regulated genes in the lipid metabolism. Skin transcriptomes of North Atlantic right whales have described diverse functions of the skin of the whales and metabolic pathways [63]. In addition, de novo blood transcriptomes have been identified in beluga whales and their functional roles have been described [64]. Studies of transcriptomes in bowhead whales have identified biological adaptations that prolong life in mammals [65]. Finally, DKK1, CEBPD, DDIT4, and ID1 have been identified as potential markers of acute hypothalamic–pituitary–adrenal (HPA) axis activation in marine mammal blubber, e.g., elephant seal [66].

Various lipid transcriptomics analyses have been conducted for farm animals. Regarding mammalian adipose tissue transcriptomes, fatty acid synthesis in pigs has been described compared to other tissues [67,68,69]. Studies of intramuscular fat (IMF) in pigs have identified potential genes and signaling pathways, such as AMP-activated protein kinase (AMPK), which plays a critical role in controlling lipid metabolism [70,71] and incRNA expression of IMF deposition [68]. In cattle, global transcriptomes in adipogenesis have given insights into the regulation of bovine adipogenic differentiation [72]. Twenty-five differentially expressed genes involved in the metabolism of lipids have been identified in chicken by transcriptomes [73]. Human adipose tissue gene expression studies have revealed the importance of environmental and individual factors in controlling the expression of human adipose tissue genes [74]. According to another transcriptome study, some adipose tissues of the Bactrian camel was shown to have functions in the immune and endocrine systems [75]. The mRNA expression patterns of dietary lipid levels controlling genes in fish give information about endoplasmic reticulum (ER) stress, unfolded protein response (UPR), and lipid metabolism [76,77,78]. Diglyceride acyltransferase (DGAT) is identified as an important catalyst in the metabolism of cellular diacylglycerol. In higher eukaryotes, such as mammals, it is involved with triacylglycerol metabolisms, such as intestinal fat absorption, lipoprotein assembly, adipose tissue formation, and lactation [79]. However, the functional and gene ontology pathways identified in other studies are linked with the lipid metabolism in particular animals, and there is no such study on marine mammals including toothed whales. Transcriptomics research on the special fats of toothed whales has the potential to identify significant genes for lipid synthesis and degradation.

Technologies on transcriptomics are still developing day by day by implementing various new tools, algorithms, and techniques. The extraction of RNA, and lipids together is an easy approach for limited tissues like from cetaceans. It has been tested in mice, lipids and associated gene expression patterns has been identified using a single sample [80]; this will be a useful approach in the future. Recent technologies have extended to high-throughput next-generation sequencing (NGS) technologies to apply in single-cell transcriptomics analyses, and biological features of organ development have been revealed, including the human heart [81]. Furthermore, the best practices in single-cell RNA-seq analysis and sample multiplexing have been reviewed and developed into an analysis pipeline [82,83]. Spatial transcriptomics is one of the newest technology introduced in the Visium platform for the identification of spatial topography of gene expression [84]. There are a few important challenges for RNA analysis, such as RNA-seq data analysis, the development of bioinformatics tools, and applications [85]. The identification of a single analysis pipeline is a major concern for the future development of transcriptomics [86]. RNA can modify due to various factors, and RNA-based modifications can happen in proteins [87], hence new developments also need to focus on epitranscriptomics, targeting the toothed whales’ metabolism. Interestingly, a dolphin blood transcriptomics study on PCBs was conducted by applying machine learning approaches and they identified changes in gene expression under the chemical exposure in the environment [33].

The transcriptome of an animal body can change due to external environmental conditions [27]. The impacts of radiation on animals’ transcriptomic levels have been studied. The potential impacts of space radiation were investigated at the transcriptomics level, concerning NF-kB pathways in different cells (mouse and human) [88]. Some cell-line studies have revealed that p53 mediated transcription modulation, induced by ionizing radiation [89,90,91,92]. Integrated global transcriptomics, and proteomic analyses have identified the induction of transforming growth factor (TGF) beta and the inactivation of peroxisome proliferator-activated receptor (PPAR) alpha signaling pathways in humans due to radiation [93]. A study on blood gene expression suggested the activation of apoptosis and the p53 signaling pathway due to ionization radiation damage by various analyses, such as differentially expressed genes analysis, weighted gene correlation network analysis, functional enrichment analysis, hypergeometric test, gene set enrichment analysis, and gene set variation analysis [94]. Further, the radiation-induced bystander effect (RIBE) has been investigated on gene expression for multiple types of RNA (mRNA, microRNA, mitochondrial RNA, long non-coding RNA, and small nucleolar RNA). Inhibition of RNA synthesis, processing, and translation can be caused by RF radiation [25]. A transcriptomics analysis on changes in lipid metabolisms of toothed whales due to radiation was not found in the searched databases. There is a high potential for radiation effects on RNA modification and changes that may cause cancers or malformations in whales. Lipid metabolism in toothed whales may involve many RNA functions; therefore, we recommend conducting this kind of lipid transcriptomics studies on toothed whales under potential environmental stressors, such as radiation.

#### 3.1.3. Proteomics

Proteomic studies on lipid droplets in different organisms can give evolutionary perspectives for different organs [95]. Compared to genomics and transcriptomics, a significant number of lipid-related proteomics studies have been conducted on toothed whales in the past. For example, LC/MS/MS has been used to profile steroid hormones in dolphin species [66]. Brown adipose tissue (BAT) of four species of delphinoid cetacean, *Lagenorhynchus obliquidens, Tursiops truncates*, *Phocoenoides dalli*, and *Phocoena phocoena* has been analyzed; the brown adipocyte-specific mitochondrial protein, uncoupling protein 1 (UCP1), is only expressed in BAT for metabolic thermogenesis [96]. Proteomes in the blubber of harbor porpoises have been studied, and functional roles, such as metabolism, immune response, inflammation, and lipid metabolism, have been identified by SDS-PAGE and nLC-ESI-MS/MS technologies [97]. Recently, the expression of arachidonic acid 12-lipoxygenating ALOX15 orthologs have been recorded in marine mammals, including toothed whales, and as a function, arachidonic acid 15-lipoxygenation was identified [98]. Another study has identified that eight toll-like receptors (TLR) signaling pathway genes were under positive selection in cetaceans; interestingly, cetacean TLR4 was less responsive to lipopolysaccharides which suggest evolutionary changes between cetaceans and ungulates [99,100,101].

Lipid metabolism-related proteomic studies have widely been conducted on other animals, too. The recent proteomics analysis of lipid droplets from an alga revealed seven novel proteins important in lipid metabolism [102]. The proteins of broiler chickens have been investigated, and creatine pyruvate (CrPyr) has been found to have a more pronounced effect on lipid and protein metabolisms than Cr or Pyr [103]. A comparative proteomics analysis has been performed with random mutants (CR12 and CR48) with altered lipids, and the study identified significant up-regulated and down-regulated proteins in microalgal lipid biosynthesis pathways [104]. The milk fat globule membrane proteins of yak exhibit high lipid accumulation-lowering efficacy and contain potential bioactive ingredients for improving metabolism [105]. Proteins related to lipid metabolism and other functions are altered by major depressive disorder mechanisms of the chronic unpredictable mild stress mouse model [106]. The induction of lipogenic genes, and proteins has been observed in Atlantic cod by monitoring the increased plasma triglyceride levels in PCB 153-treated fish [107]. However, integrated proteomics on lipid metabolism of toothed whales are still lacking; therefore, the above findings may give insight into the future studies.

Lipidomics and proteomics analyses to find structural changes and species can easily be done with recent LC/MS or GC/MS technologies. The LIPID MAPS Proteome Database is useful for identifying lipid-associated protein sequences and annotations, and makes it possible to develop lipid interaction networks and integrate them with lipid metabolism pathways [108]. A high-resolution isotomic approach has been tested in a study to identify stable isotopes in the mammalian metabolism, including cetaceans, and they identified that extensive 13C enrichment was likely associated with fasting in the humpback whale [109]. Machine learning has been applied to proteomics to identify suitable biomarkers of diseases and predict proteins for treatment [110].

Radiation proteomics is an interesting field to discover molecules of radiation, biomarkers, acute and chronic physiological and health effects under different levels of exposure [111]. They have identified that ionizing gamma radiation could deregulate numerous proteins. Therefore, radiation can affect the function, structure, modifications, and interactions of proteins in an animal body. Under an integrated proteome and miRNA study, a decrease in the miR-21 level was observed and it was affected to alter several target proteins [112]. The fatty acid-binding protein 5 (FABP5) was investigated in human skin, and the increased level was investigated with intensifying TGF-β signaling pathway due to radiation [113]. Enhanced retinoic acid-induced signaling of PPARβ/δ and DHA-induced activation of RXR were also identified as an effect of radiation [114]. A similar study revealed that radiation-induced skin fibrosis was enhanced due to an overexpression of ZIP9 via DNA demethylation by radiation [115]. Another study of mitochondrial proteomics investigated a mechanism of apoptosis of spermatogenic cells in zebrafish caused by radiation [116]. Several technologies are currently famous for proteomics studies on radiation, such as ICPL, iTRAQ, and SILAC; however, comparative proteomics with bioinformatics is recommended for deep study on biological changes [117]. RF radiation causes denaturation of proteins and may act as a stressor to overexpress heat-shock proteins harmfully [62]. Proteome analysis of serum, and plasma has advantages for studies of radiation biology, and biomarker discovery [118]. In contrast, there is a huge gap of knowledge on proteomics of lipid metabolism, and radiation on toothed whales. Therefore, in the future, scientists should focus on addressing this gap by identifying potential radiation-related threats and their impacts. Accordingly, these findings will be useful in sustainable decision making to conserve these large animals in the ocean environment.

#### 3.1.4. Lipidomics

Lipidomics is the comprehensive study of fats, and their components in the cells of organisms and also of the different biosynthetic pathways [119]. There are eight categories of lipids and more than 1 million lipid species [120]. In invertebrates, fats play a major role in energy storage and homeostasis; however, in vertebrates, fats are involved with many other functions such as immunity, protein synthesis, and metabolism [12]. Lipidome can affect the maximum lifespan of a species; however, identification of these lipidome determinants of a species’ maximum lifespan is still at the development stage [121]. Lipids can be utilized for quantitative diagnosis, monitoring treatment response, and patient stratification as peripheral biomarkers [122].

During the past five centuries, research interest in lipids in toothed whales has gradually increased compared to genomics, transcriptomics, and proteomics. In toothed whales, several fat bodies have been identified. The development of the melon in toothed whales is still unclear; however, biochemical analysis has revealed that a portion of the isovalerate differs with the development of the body [123]. Mandibular fats of toothed whales perform acoustic functions through the expression of branched-chain fatty acids, which can alter the properties of the sound [8]. On the other hand, GC/MS, and HPLC techniques have identified high levels of fatty acid stratification in the outer blubber rather than the inner blubber in white whales, and killer whales [124]. Koopman has emphasized the necessity to conduct integrated omics studies to achieve a clear understanding of the lipid metabolism in toothed whales [11]. According to an ingested lipids analysis, right whales have evolved an unusual metabolic capability [14]. The fatty acid composition of the blubber of minke whales was investigated, and evidence of high metabolic activity was provided by the presence of long-chain polyunsaturated fatty acids (PUFAs) [125].

Lipid synthesis in many different organisms has been investigated. Triacylglycerol esters store metabolic energy as fats, and the synthesis of TAG was described 50 years ago; however, more research is needed to complete the understanding of the molecular mechanisms of TAG synthesis [126]. For example, in corals, lipogenesis in lipid bodies are controlled by coral hosts and metabolites such as triglycerols, sterol esters, and free fatty acids [127]. Further, the cell membrane is an important region for the study of lipid and protein interactions [128]. Phospholipid separation with high resolution, specificity, and signal-to-noise ratio can be done via Search Results DMS (Differential Mobility Spectrometry) with the combination of current liquid chromatography (LC) methods [129]. Microbial productions of triacylglycerols or fatty acid ethyl esters are concerned, these days, as fuel replacements or other compounds [130]. A recent study confirmed that information about wax ester metabolism was obtained by a simple gene (ADP1) insertion in bacteria [131]. Wax ester metabolism is also an important topic to discuss, especially in toothed whales.

Lipidomics is a part of metabolomics, a still-developing field with many techniques to study various types of lipid species that have unique characteristics [132]. The Folch method is the widely used lipid extraction protocol for animal tissues [133]. A newly introduced butanol and methanol (BUME) method has several advantages: simplicity, throughput, automation, solvent consumption, lower cost, health, and environmental aspects, over the Folch method for lipid extraction [134]. The use of greener solvents for lipid extractions is also considered to be in the development stage [135]. According to recent advances in lipid-related technology, two approaches have been adopted to understand the utility of sphingolipidomics, such as LC-MS- or LC-MS/MS-based, and shotgun lipidomics-based approaches [136,137]. New pulse programs have been developed for measuring lipids by nuclear magnetic resonance (NMR) [138] or HR-MAS NMR [139]. The lipid composition can vary significantly between cells, tissues, and organs; therefore, there is a need for technology with high mass accuracy, high resolution, and space atmospheric pressure, such as reliable and reproducible methods, such as MALDI MSI, for the identification of lipids [140]. Cryo-electron microscopy, and X-ray crystallography are also techniques for observing the interaction of lipids and membrane proteins [141]. Ultra-high performance liquid chromatography high-resolution mass spectrometry (UHPLC-HRMS) is a novel technology for lipidome analysis [119]. Another approach involves combining hydrophilic interactions (HILIC) with C30 reversed-phase chromatography (C30RP) coupled to high-resolution mass spectrometry (HRMS) as high-throughput platforms to analyze complex lipid mixtures [142]. Other ways are ‘top-down’ lipidomics, and ‘bottom-up’ lipidomics (Shevchenko and Simons, 2010). Flow field–flow fractionation (FlFFF)-MS is a high-speed technique for the analysis of lipoproteinic lipids as a top-down method [143]. Nanoflow liquid chromatography-ESI-MS/MS (nLC-ESI-MS/MS) analysis is a novel approach for a bottom-up approach. Lipidomics has generated a large amount of data; however, there is a need for bioinformatics and statistics for meaningful analysis. The development of chromatography and mass spectrometry are driving ongoing improvements in analytical performance to identify lipid species [144]. These researchers also noted the inadequacy of a single analytical platform for metabolomics analysis and, therefore, described the application of multi-omics approaches. Such approaches should be integrated with methods, and technologies, extraction protocols, and molecular systems-based analytical, and bioinformatics tools [120].

Understanding lipid dynamics and the role of enzymes in the metabolism pathway in toothed whales is still a challenge. Recently, scientists have looked at the structures, functions, interactions, and dynamics of single cellular lipids; therefore, the integration of lipidomics data with multi-disciplinary information is necessary for the analysis of metabolic pathways [145]. Lipids are important for membrane dynamics, energy homeostasis, and regulation of the molecular machinery, which means that lipids are a source of biological information, and biomarkers for cancer studies [146]. Lipid biomarkers are also becoming familiar in many fields and the adipocytes index in humpback whales is considered a non-destructive biomarker [147]. Therefore, integrated lipidomics has a potential to identify novel biomarkers in toothed whales in the future.

Lipids are sensitive molecules for environmental science studies since their abundance in any form of life and the lipid profiles can easily change with external stimuli [132,148], such as radiation. Metabolomic studies on the effects of radiation have been widely done; however, in this review, we especially focused on lipidomics studies for radiation effects. Exposure to radiation may cause reactive oxygen species (ROS) and damage cellular lipids [149]. Another lipidomics study found that oxidized phospholipids by radiation produce apoptotic signaling, causing lung damage in animals [150], emphasizing redox lipidomics, a growing field for the study of programmed cell death—apoptosis and ferroptosis [151]. Modifications of radiation effects can be observed by lipid peroxidation [152]. Radiation biodosimetry is another developing field and radiation-responsive lipids biomarkers identified in lipidomics studies are important in this development [153]. A recent study has investigated seven radiation-responsive lipids, including PC (18:2/18:2), PC (18:0/18:2), Lyso PC 18:1, PC (18:0/20:4), SM (D18:0/24:1), PC (16:0/18:1), and Lyso PC 18:2 which are useful in radiation biodosimeters [154]. In this study, they have observed that ionizing radiation causes changes in phospholipid metabolism by increasing the amount of phosphatidylethanolamine (PE) and phosphatidylserine (PS). The biological actions of n-3 PUFA (Omega-3 polyunsaturated fatty acids) under UV radiation has been investigated and its potential to use as a nutrient for skin health has been revealed [155]. Effects of radiation on damaging lipid profiles in cancer patients have been investigated under laboratory conditions; however, there is still more to do regarding a study on the effects of radiation on other wild animals in nature [156]. Recently, potential lipid biomarkers (distributed in linoleic acid metabolism, glycerophospholipid metabolism, glycerolipid metabolism, and glycosylphosphatidylinositol (GPI)-anchor biosynthesis) were identified as a result of microwave radiation; therefore, lipid metabolism studies under radiation impacts give insight into future research targets [18]. Based on many lipidomics and radiation studies, it is evident that various types of radiation have the potential to change lipid profiles in animals and damage the natural metabolism. Therefore, we can predict that there is a high potential for the impact of radiation on toothed whales’ lipid metabolism. We recommend qualitative and quantitative measurements of lipid molecules under radiation exposure as a promising technique for lipidomics. Comparative lipidomics studies of toothed whales’ populations in different healthy and unhealthy environments may also give useful information to identify the effects of environmental stressors on their lipids.

#### 3.1.5. Integration of Multi-Omics

Many studies in the past have suggested that the integration of several omics’ platforms is suitable for lipid metabolism analysis for different purposes (Supplementary Table S1). DNA or RNA isolation and sequencing have progressively become a component of multi-omics approaches, in which several layers of biology are simultaneously captured and analyzed. Multi-omics has many advantages, such as data integration and modeling pipelines, to predict metabolic pathways in animal metabolism. We believe that multi-omics is a highly potential approach to elucidate unique fatty acid synthesis and metabolism in toothed whales. For example, a recent study investigated the role of coiled-coil domain-containing protein 80 (CCDC80) gene in lipid metabolism by applying multi-omics integrative analysis and they found new roles of CCDC80 in fatty acid metabolism [157]. Moreover, multi-omics has been widely used for identifying molecular and non-molecular biomarkers [158,159]. Therefore, another benefit of large-scale multi-omics is that it may predict lipid biomarkers, and it can be useful in precision medicine. Omics data generation and integration are enhancing, in molecular biology and biotechnology, discoveries, such as new biomarkers. One of the driving forces behind the emergence of multi-omics approaches is an increasing appreciation of the contribution of multiple regulatory pathways and paradigms and of their relevance to processes that govern development, health, and disease. The combined studies of genetics, and transcriptomics have become popular in human medicine, e.g., for diagnosis and treatment of systemic lupus erythematosus [160]. In precision medicine, the integration of genomics, proteomics, and metabolomics has been identified as a potential approach for the robust characterization of biochemical signatures reflective of organismal phenotypes [161].

The combination of several omics platforms has been investigated in different animals successfully. In the arthropod metabolism, genes control the wax-secreting organ; therefore, proteins, and pathways in the lipid metabolism have been identified in ticks by transcriptomes, and proteomes [162]. Large amounts of omics data have been generated around the world; therefore, a global-integrative analytical approach needs to be established for future research [163]. Basic ML techniques, such as neural network analysis, have been widely used in studies of cetaceans for different purposes [164,165]. A combination of ML with multi-omics data may uncover relationships between molecules [166]. Therefore, we believe that the use of this combination of technologies with ML could help in finding the hidden pathways of complex lipid metabolism in toothed whales.

Applying multi-omics on the biological impacts of radiation is still an innovative field of study. A recent investigation experimentally revealed that the gut microbiota-metabolic axis acts as a shield for radiation in the animal body using the multi-omics approach, and this microbiota is important in producing the short-chain fatty acids that are used in various metabolic reactions in the host [167]. Several multi-omics techniques, such as RNA-seq, exome-seq, and H3K27ac ChIP-seq, were tested in a study to investigate the effects of UV radiation on human skin homeostasis and they found global gene dysregulation in skin cells under UV radiation [168]. Another non-animal study found that rice seed has adapted its biology to the low-level radioactive environment by multi-omics analysis [169]. The use of a combined approach of multi-omics to identify radiation effects on cetacean, including toothed whales, has not been conducted. Therefore, in this review, we also suggest focusing research on radiation by linking genomics, transcriptomics, proteomics, metabolomics, epigenetics, and environmental genomics applications to understand the impacts of radiation on lipids in toothed whales comprehensively.

### 3.2. Acoustic Radiation on Lipid Metabolism in Toothed Whales

From microorganisms to large animals/plants, every living organism consists of lipids, and individual lipid profiles can be changed by environmental stress [132]. Lipids play an important role in toothed whales’ echolocation; however, the combination and profile of these specialized lipids may interact with different stressors that may cause misbehaviors. Several environmental stressors include depletion in ozone, increasing UV radiation, loss of biodiversity, and temperature [170]. Different types of radiation, such as isotopic, electromagnetic, and shortwave, have been largely discussed on biological aspects. Therefore, in this review, we selected acoustic radiation as a high impact environmental stressor to toothed whales.

A study found that low-intensity pulsed ultrasound (LIPUS) can cause visceral adipocyte differentiation in an experimental manner [171]. One study investigated that shock waves can interact with lipid membranes and, therefore, they used this technique for drug delivery by Lipid-mRNA nanoparticle [172]. Another approach is the use of acoustic radiation to investigate lipids for therapeutic purposes. Lipid particles, such as bubble liposomes, have been investigated for gene and drug delivery in the presence of ultrasound acoustic radiation [173]. The role of microwave radiation in changing lipid metabolism has also been studied recently, providing new therapeutic strategies [18]. The acoustic radiation force impulse imaging (ARFI) is a concept that is currently practiced as a liver biomarker to assess liver fibrosis, which produces shear waves in damaged tissue for monitoring [174]. Invertebrates, crustaceans, fish, and marine mammals produce sounds in a range of frequencies for foraging, mating, and complex communications naturally in water [175]. However, due to the natural and anthropogenic underwater noises, a shrinking communication space in whales has been observed [176]. Underwater noise pollution and its effects on whales have been widely discussed. For example, this noise may cause auditory masking, behavioral changes, hearing damage, and death for marine mammals, as they are very sensitive to acoustic radiation [177]. Therefore, it is evident that underwater noises, and acoustic radiation pollution should have a potential impact on sensitive tissue alterations in underwater animals, especially large animals, such as cetaceans. Currently, information is emerging on how acoustic radiation or underwater noise influence lipid metabolism in animals.

There are limited data on how metabolic alterations can happen in the animal body due to the noise pollution. Impacts of non-ionization radiation on lipids can be elucidated in laboratory condition using cetaceans’ cell-lines, such as fibroblasts, adipocytes, and blood samples. In the natural environment, more comprehensive studies, including novel omics approaches, are needed in the future to investigate the effects of acoustic pollution on specialized lipid metabolism and to understand echolocation misbehaviors in toothed whales. However, understanding the whole process of these changes is more complicated to recognize on an individual level, and, therefore, integration of different omics-based studies can contribute significant results for making predictions and management decisions. ML and DL have been used for detection of acoustic sounds in cetaceans for identification purposes [178,179,180]. Therefore, recent ML tools may have clear potential to detect acoustic radiation impacts on toothed whale by detecting changes from their normal behaviors. ML is also useful for problem-solving in healthcare, and radiation oncology [181]. Therefore, this review also suggests implementing machine learning approaches for further understanding the lipid metabolism in toothed whales under environmental stresses, such as underwater acoustic radiation pollution. In the natural environment, establishment of this kind of study needs more innovative techniques. Molecular tagging methods has some potential to develop into a data collection tool in applying multi-omics on investigating acoustic radiation impacts in toothed whales in the wild.

## 4. Conclusions

Aquatic environments in the world are highly threatened by various natural and anthropogenic factors; therefore, understanding the interactions between animals and their environment, and possible stressors are highly important for sustainable ecosystem management. In this review, we evaluated lipid-related studies that have applied omics technologies and impacts of radiation to elucidate lipid metabolism in toothed whales. We identified that lipidomics, and proteomics use similar technologies, such as LC/GC/MS, while genomics, and transcriptomics also use the same technologies, such as NGS. The use of small single sample for various omics approaches has rarely been a concern in cetacean research, and, therefore, it has high potential to generate various big data for integrative analysis. There are examples for single sample preparation for lipidomics and transcriptomics analyses, which will be important for studies in toothed whale species due to the difficulties of finding fresh samples. The combination of omics approaches in toothed whales’ lipid metabolisms is very limited and we identified it as a research gap in this review. Multi-omics approaches are useful to study the environmental effects on lipid metabolism in toothed whales due to their complexity. We discussed the effects of radiation as an environmental stressor that may change the lipid profiles of toothed whales. Radiation effects, including acoustic pollution, on toothed whales and their specialized lipid metabolism changes have been not studied largely. Therefore, this review suggests applying omics techniques and machine learning in this kind of research to identify mechanisms on alteration of lipid metabolism in toothed whales by external stressors. Considering all the aspects, an integrated multi-omics application will be the most reliable approach for the future studies of the complex lipid metabolism of toothed whales and to understand the impacts of radiation on their lipid profile changes in the underwater environment.

## Data Availability

No new data were created or analyzed in this study. Data sharing is not applicable to this article.

## Supplementary Materials

The following are available online at https://www.mdpi.com/article/10.3390/life11040364/s1, Supplementary Table S1. Reference list used for understanding future approaches to understand lipid metabolism in toothed whales.
