# Supplementary material for: Multi-Omics Approaches and Radiation on Lipid Metabolism in Toothed Whales
Source: Life (Basel). 2021 Apr 20;11(4):364. doi: 10.3390/life11040364 (PMC8074237; doi:10.3390/life11040364)
Supplement: Supplementary file 1 [file life-11-00364-s001.zip › life-1162260-supplementary.pdf]

life

Review

Multi-Omics Approaches and Radiation on Lipid Metabolism in Toothed Whales

Jayan D. M. Senevirathna [1,2,*] and Shuichi Asakawa [1]

1 Laboratory of Aquatic Molecular Biology and Biotechnology, Department of Aquatic Bioscience, Graduate School of Agricultural and Life Sciences, The University of Tokyo, Tokyo 113-8654, Japan; asakawa@g.ecc.u-tokyo.ac.jp
2 Department of Animal Science, Faculty of Animal Science and Export Agriculture, Uva Wellassa University, Badulla 90000, Sri Lanka
* Correspondence: duminda@uwu.ac.lk

Abstract: Lipid synthesis pathways of toothed whales have evolved since their movement from the terrestrial to marine environment. The synthesis and function of these endogenous lipids and affecting factors are still little understood. In this review, we focused on different omics approaches and techniques to investigate lipid metabolism and radiation impacts on lipids in toothed whales. The selected literature was screened, and capacities, possibilities, and future approaches for identifying unusual lipid synthesis pathways by omics were evaluated. Omics approaches were categorized into the four major disciplines: lipidomics, transcriptomics, genomics, and proteomics. Genomics and transcriptomics can together identify genes related to unique lipid synthesis. As lipids interact with proteins in the animal body, lipidomics, and proteomics can correlate by creating lipid-binding proteome maps to elucidate metabolism pathways. In lipidomics studies, recent mass spectroscopic methods can address lipid profiles; however, the determination of structures of lipids are challenging. As an environmental stress, the acoustic radiation has a significant effect on the alteration of lipid profiles. Radiation studies in different omics approaches revealed the necessity of multi-omics applications. This review concluded that a combination of many of the omics areas may elucidate the metabolism of lipids and possible hazards on lipids in toothed whales by radiation.

Keywords: genomics; transcriptomics; proteomics; lipidomics; radiation; cetaceans

Citation: Senevirathna, J.D.M.; Asakawa, S. Multi-Omics Approaches and Radiation on Lipid Metabolism in Toothed Whales. *Life* **2021**, *11*, 364. https://doi.org/10.3390/life11040364

Academic Editor: Fabrizio Ambrosino and Supitcha Chanyotha

Received: 13 March 2021
Accepted: 17 April 2021
Published: 20 April 2021

Publisher's Note: MDPI stays neutral with regard to jurisdictional claims in published maps and institutional affiliations.

Copyright: © 2021 by the authors. Submitted for possible open access publication under the terms and conditions of the Creative Commons Attribution (CC BY) license (http://creativecommons.org/licenses/by/4.0/).

Supplementary Materials:

Table S1. Reference list used for understanding future approaches to understand lipid metabolism in toothed whales.

No	Reference	Title	Future Focus
GN1	[1]	Genetic Regulation of Plasma Lipid Species and Their Association with Metabolic Phenotypes	To Identify genes and genetic variants associated with plasma lipid species to enhance understanding of biochemical pathways. To facilitate the design of new therapies for metabolic diseases.
GN2	[2]	Expression of Genes Involved in Lipid Metabolism Correlate with Peroxisome Proliferator-Activated Receptor Y Expression in Human Skeletal Muscle	To find unknown or unmeasured factors that influence PPAR-g and other measured transcripts.
GN3	[3]	Mining the genome for lipid genes	To prioritize and develop high-throughput functional assays. To reduce generation of more data and use the existing GWAS data deeply concerning. To find new bioinformatics approaches of use of other omics data to identify and/or prioritize candidate genes.
GN 4	[4]	A genomics approach reveals insights into the importance of gene losses for mammalian adaptations	To uncover the molecular mechanisms underlying the evolution of a wide range of adaptive phenotypes, which will deepen our understanding of how nature's fascinating phenotypic diversity has evolved
GN 5	[5]	Return to the Sea, Get Huge, Beat Cancer: An Analysis of Cetacean Genomes Including an Assembly for the Humpback Whale (*Megaptera novaeangliae*)	To consider comparative oncology research that seeks to improve therapeutic targets for human cancers. To provide a resource for developing useful genomic markers that will aid in the population management and conservation of whales.
GN6	[6]	One reference genome is not enough	To improve the quality of future short-read alignments and variant calling.
GN7	[7]	Loss of inherited genomic imprints in mice leads to severe disruption in placental lipid metabolism	To determine what effects are primarily due to a defect in genomic imprinting and which effects are secondary. To find important changes in the transcription of genes implicated in triacylglycerol trafficking. To assess protein concentrations or post-translational protein modifications.
GN8	[8]	Beyond thermoregulation: metabolic function of cetacean blubber in migrating bowhead and beluga whales	To expand the existing knowledge and offer unique insight into how the regulation of *Lep* and lipolysis has adapted to permit seasonal deposition and maintain vital blubber stores.
GN9	[9]	Genetic Signatures of Lipid Metabolism Evolution in Cetacea	To study genes and the associated pathways for future investigations of cetacean lipid metabolism.
GN10	[10]	Identification of the Wax Ester Synthase/Acyl-Coenzyme A:Diacylglycerol Acyltransferase WSD1 Required for Stem Wax Ester Biosynthesis in Arabidopsis[1,2][W][OA]	To consider WSD1, a member of the Arabidopsis WS/DGAT enzyme family that catalyzes the biosynthesis of wax esters for wax production.
GN11	[11]	An integrative systems genetic analysis of mammalian lipid metabolism	To give opportunities for the discovery of therapeutic agents and biomarkers in the setting of

			hepatic lipotoxicity. To use PSMD9 as a regulator of lipid metabolism, with potential therapeutic implications. To serve datasets and methodologies to both complement and validate existing and forthcoming discovery resources.
GN12	[12]	'Obesity' is healthy for cetaceans? Evidence from pervasive positive selection in genes related to triacylglycerol metabolism	To investigate functional verification of TAG metabolism-related genes in cetaceans and other marine mammals. To determine their role in aquatic adaptations. To study TAG metabolism during their adaptation to various environments, and to interpret the roles of TAG metabolism in terrestrial mammals.
TP1	[13]	Identification of a gene encoding an acyl CoA:diacylglycerol acyltransferase, a key enzyme in triacylglycerol synthesis	To use this cDNA encoding a DGAT in studies of cellular glycerolipid metabolism and its regulation
TP2	[14]	Dual extraction of mRNA and lipids from a single biological sample	To use in both transcriptome and lipid analysis in high-throughput analysis of metabolic diseases, such as NAFLD. To analyze biopsies to explore both the transcriptome and lipidome, especially when the amount of tissue is limited, and provide novel opportunities for multi-omic data analysis through sample-specific network analysis.
TP3	[15]	Identification of five genes in endoplasmic reticulum (ER) stress–apoptosis pathways in yellow catfish *Pelteobagrus fulvidraco* and their transcriptional responses to dietary lipid levels	To analyze relations between ER stress and lipid metabolism in fish to understand potential role and mechanism of apoptosis induced by ER stress.
TP 4	[16]	Blubber transcriptome response to acute stress axis activation involves transient changes in adipogenesis and lipolysis in a fasting-adapted marine mammal	To validate additional markers using an increased sample size and to determine if these markers, and whole-transcriptome responses in general, differ between acute and chronic stress states.
TP 5	[17]	Analysis of porcine adipose tissue transcriptome reveals differences in de novo fatty acid synthesis in pigs with divergent muscle fatty acid composition	To study muscle transcriptome in pigs, together with liver and adipose tissue transcriptomes, in order to obtain a complete view of FA metabolism.
TP 6	[18]	Sampling the skin transcriptome of the North Atlantic right whale	To study multiple physiological processes including those related to immunity and stress response.
TP 7	[19]	De novo transcriptome assembly and RNA-Seq expression analysis in blood from beluga whales of Bristol Bay, AK	To establish beluga blood transcriptome concerning the impacts of season, sexual maturity, disease, and geography. To provide wealth of transcriptomic data on beluga whales and preliminary data for comparison with other studies in beluga whale.
TP 8	[20]	Single-Cell Transcriptome Analysis Maps the Developmental Track of the Human Heart	To use the mechanisms of in vivo human cardiac development and provide potential data on cardiac regeneration.

TP 9	[21]	The transcriptome of the bowhead whale *Balaena mysticetus* reveals adaptations of the longest-lived mammal	To provide bowhead whale transcriptome date for further research, including genome annotation and analysis, the evolution of longevity, adaptation to an aquatic environment, conservation efforts and as a reference for closely related species.
TP 10	[22]	Data Mining and Validation of AMPK Pathway as a Novel Candidate Role Affecting Intramuscular Fat Content in Pigs	To interpret the different molecular mechanisms of IMF deposition between lean and fat pig breeds
TP 11	[23]	Analysis and preliminary validation of the molecular mechanism of fat deposition in fatty and lean pigs by high-throughput sequencing	To provide a basis for studying the different metabolic lncRNA expression of IMF deposition. To study the mechanisms of obesity, pigs may represent a new avenue for studying human obesity. To provide a reference for studying lncRNAs in other species.
TP 12	[24]	Global Transcriptome Analysis During Adipogenic Differentiation and Involvement of Transthyretin Gene in Adipogenesis in Cattle	To study an array of new questions related to the molecular mechanisms underlying the regulation of bovine adipocyte differentiation and provide primary information for further functional studies on TTR in bovine adipogenesis.
TP 13	[25]	Current best practices in single-cell RNA-seq analysis: a tutorial	Two avenues of development that are of particular interest due to their potential for disruption to analysis pipelines are deep learning workflows and single-cell omics integration.
TP 14	[26]	A comparative analysis of the transcriptome profiles of liver and muscle tissue in pigs divergent for feed efficiency	To study increased expression of the genes associated with this gene ontology term suggest that the more efficient (LRFI) pigs have improved capacity to metabolise fats. To find common pathways across tissues as well as tissue specific pathways that contribute to differences in feed efficiency.
TP 15	[27]	MULTI-seq: sample multiplexing for single-cell RNA sequencing using lipid-tagged indices	To use MULTI-seq for sample barcodes and to reveal the mechanisms by which cell populations interact to promote development, homeostasis and disease.
TP 16	[28]	Advanced Applications of RNA Sequencing and Challenges	To improve computational modeling and algorithm designs beyond the existing ones by applications from NGS to nanopore sequencing and single-cell sequencing.
TP 17	[22]	Data Mining and Validation of AMPK Pathway as a Novel Candidate Role Affecting Intramuscular Fat Content in Pigs	To study regulatory function of the AMPK signaling pathway in relation to the IMF content in the LD muscle of different pigs. To interpret the different molecular mechanisms of IMF deposition between lean and fat pig breeds.
TP 18	[29]	Determinants of Human Adipose Tissue Gene Expression: Impact of Diet, Sex, Metabolic Status, and Cis Genetic Regulation	To apply genomics technology to dietary intervention aimed at maintaining weight loss.

TP 19	[30]	RNA-Seq Analysis of Abdominal Fat in Genetically Fat and Lean Chickens Highlights a Divergence in Expression of Genes Controlling Adiposity, Hemostasis, and Lipid Metabolism	To find bioinformatics analyses for further definitive studies to verify the importance in lipogenesis and adipogenesis, and to gain mechanistic insight into genetic control of adiposity in the chicken.
PT1	[31]	LMPD: LIPID MAPS proteome database	To develop lipid interaction networks that will integrate lipid metabolic pathways and signaling networks, and tools for exploring these networks. To study biological processes underlying lipid-involved disease processes and lead to the identification of potential drug targets.
PT 2	[32]	Application of Machine Learning to Proteomics Data: Classification and Biomarker Identification in Post-genomics Biology	To analyze proteins identified as possible biomarkers. To conduct pathway analysis to verify relationships between proteins found by machine learning.
PT 3	[33]	Not just fat: investigating the proteome of cetacean blubber tissue	To assign novel functions to marine mammal blubber in keeping with current understanding of the multifunctional role of adipose tissue in other mammals. To develop biomarkers to better monitor the physiological state and health of live individuals though remote blubber biopsy sampling. To identify key metabolic processes and pathways and therefore assign novel functional roles to marine mammal blubber tissue.
PT 4	[34]	The proteomics of lipid droplets: structure, dynamics, and functions of the organelle conserved from bacteria to humans	To study lipid droplets from a broad range of sources, facilitating further investigations of their cellular functions and roles in the pathology of metabolic diseases as well as in bio-fuel development.
PT 5	[35]	Proteomics Analysis of Lipid Droplets from the Oleaginous Alga *Chromochloris zofingiensis* Reveals Novel Proteins for Lipid Metabolism	To study algal LD biogenesis and TAG synthesis to facilitate future genetic engineering of industrially applicable algae for improvements in TAG and other storage compounds.
PT 6	[36]	Use of comparative proteomics to identify the effects of creatine pyruvate on lipid and protein metabolism in broiler chickens	To study some key proteins that help limit obesity-related problems during production and exploring new ways to improve muscle growth in broilers.
PT 7	[37]	Comparative proteomics using lipid over-producing or less-producing mutants unravels lipid metabolisms in *Chlamydomonas reinhardtii*	To reveal various potential genes or pathways directly or indirectly linked to microalgal lipid production.
PT8	[38]	Label-free quantitative proteomic analysis of milk fat globule membrane proteins of yak and cow and identification of proteins associated with glucose and lipid metabolism	To use bioinformatics analysis of proteomics can really predict the possible bioactivities, and yak and cow MFGM proteins

PT9	[39]	Quantitative proteomics analysis of the liver reveals immune regulation and lipid metabolism dysregulation in a mouse model of depression	To investigate limitations to provide further detail and verify more proteins.
PT10	[40]	Quantitative proteomics analysis reveals perturbation of lipid metabolic pathways in the liver of Atlantic cod (*Gadus morhua*) treated with PCB 153	To use integrated analysis of transcriptomics and proteomics datasets.
LP1	[41]	Obesity and the regulation of fat metabolism	To find the energy balance of the mammalian homologs of fat genes identified in *C. elegans*.<br>For understanding fat regulation by multiple layers of investigation spanning from metabolism, transcription and signaling to neuronal development and behavior.
LP2	[42]	Triacylglycerol synthesis and energy metabolism: a gut reaction?	To find the molecular mechanisms by which inhibiting intestinal TG synthesis increases energy expenditure.
LP3	[43]	Three-dimensional enhanced lipidomics analysis combining UPLC, differential ion mobility spectrometry, and mass spectrometric separation strategies	To use methodologies along with RP chromatography that allow full isobaric resolution, identification, and compositional characterization of specific phospholipids at the molecular level.
LP4	[44]	Characterization of lipids in adipose depots associated with minke and fin whale ears: Comparison with "acoustic fats" of toothed whales	To find different lineages of odontocetes which may have subsequently acquired the ability to synthesize and deposit wax esters and short, branched fatty acids (FA) as they specialized in echolocation and ultrasonic hearing.
LP5	[45]	Qualitative and quantitative study of the highly specialized lipid tissues of cetaceans using HR-MAS NMR and classical GC	To use HR-MAS NMR, which provides a global lipid composition of the sample using very little material, in a short-time frame, and without any sample preparation.
LP6	[46]	Optimization of Folch, Bligh-Dyer, and Matyash sample-to-extraction solvent ratios for human plasma-based lipidomics studies	To use multi-omic capability for analysis of multiple volumes of plasma, trace metabolite and lipid species, and additional analyte classes.
LP7	[47]	Assessment of bacterial acyltransferases for an efficient lipid production in metabolically engineered strains of *E. coli*	To use a set of 10 acyltransferases, especially AtfA that are suitable for promoting high TAG synthesis, while the mutated version AtfA-G355I, high FAEE production in *E. coli*.
LP8	[48]	Recent Analytical Trends in Lipidomics; Techniques and Applications in Clinical Medicine	To find dynamic lipidomics for estimating lipid turnover kinetics and the effects of individual enzyme deactivation or activation on lipid homeostasis.<br>To apply single cell lipidomics that can characterize the unique chemical composition of individual cells, and is the new area of advancing lipidomics.
LP9	[49]	Training in metabolomics research. I. Designing the experiment, collecting and extracting samples and generating metabolomics data	To use new pulse programs that have been developed for measuring lipids by NMR.<br>To apply statistical methods to metabolomics data, identification of metabolites, and the pathways of

			metabolism. To try new areas of application.
LP 10	[50]	Rapid and reliable steroid hormone profiling in *Tursiops truncatus* blubber using liquid chromatography tandem mass spectrometry (LC-MS/MS)	Analyzing pathway alterations in hormone physiology through this methodology, using a single sample, can provide more in-depth endocrine health information, which is valuable for the monitoring and health of these protected species.
LP 11	[51]	Mass Spectrometry Methodology in Lipid Analysis	To address four main issues; firstly, the sample pretreatment procedures, including collection, transportation, conservation and extraction, need to be standardized; secondly, the analytical methods and data obtained have to be cross-validated in different laboratories; thirdly, analytical approaches for the accurate analysis of some lipids, such as gangliosides, phosphoinositides, pregnenolone, etc., are still lacking; fourthly, to improve the data interpretation and the informatics technologies to attain meaningful biological information from lipid data.
LP 12	[52]	Lipidomics: coming to grips with lipid diversity	To introduce quantitative lipidomics into ongoing research in different model systems, for example yeast, *Drosophila melanogaster*, *Caenorhabditis elegans*, *Danio rerio* and mice, and transform the now restricted niche of lipid research into a multi-disciplinary systems biology platform. To apply Powerful Reductionist Approaches, and combine reductionist biology with systems approaches.
LP13	[53]	Brown Adipose Tissue in Cetacean Blubber	To find that fat bodies composed of typically mammalian lipids may also be acting as "acoustic" fats in some mysticete cetaceans. To investigate potentially important functions of the fatty tissue.
LP 14	[54]	Lipidome determinants of maximal lifespan in mammals	To identify presence of a distinct molecular mechanism controlling maximum lifespan variation among mammalian species.
LP 15	[55]	Coral lipid bodies as the relay center interconnecting diel-dependent lipidomic changes in different cellular compartments	To investigate the role of lipids trafficking and monitor the lipidomics of host gastrodermal cells under various photosynthetic states.
LP 16	[56]	Targeting Modified Lipids during Routine Lipidomics Analysis using HILIC and C30 Reverse Phase Liquid Chromatography coupled to Mass Spectrometry	To combine chromatography as a complementary platform with high resolution tandem mass spectrometry allowing excellent resolution of lipid isomers or isobars, and accurately distinguish di/triglycerides, plasmalogens and ether isoforms of lipids in a diverse mix of biological samples.
LP 17	[57]	Large-scale human skin lipidomics by quantitative, high-throughput shotgun mass spectrometry	To use absolute quantification and high-throughput shotgun mass spectrometry for rigorous and systematic studies of influence of drugs on the skin lipidome; the action of substances influencing skin lipid metabolism or the skin microbiome-lipidome relation; impact of cosmetic substances on a skin lipidome.
LP 18	[58]	Distribution and development of the	Combining analyses of the biochemical

		highly specialized lipids in the sound reception systems of dolphins	compositions and spatial arrangements of acoustic fat bodies with acoustic behavior studies would allow us to greatly improve our understanding of exactly how these remarkably specialized adipose tissues develop and function.
LP 19	[54]	High-resolution atmospheric pressure MALDI mass spectrometry imaging workflow for lipidomics analysis of late fetal mouse lungs	To perform quantitative analysis of lipids and other cellular metabolites from different tissue sections using MALDI MSI.
LP 20	[59]	Mammalian Triacylglycerol Metabolism: Synthesis, Lipolysis and Signaling	To identify novel crosstalk between TAG metabolism and additional processes or pathways, its relationship to metabolic diseases such as obesity, diabetes and cardiovascular disease. To study mammalian enzymes of TAG synthesis in a purified state without epitope tags, recognize and interact with their substrates and release their products, orientation within membranes, and interaction with other proteins.
LP 21	[60]	Liquid Chromatography Techniques in lipidomics Research	To describe lipid molecular species, present in AT for LC–MS methods comparison and for integration of lipidomics data in systems biology and medicine workflows with high predictive and diagnostic potential.
LP 22	[61]	Real-Time monitoring of intracellular wax ester metabolism	To employ more extensive and wide-ranging experimental set-ups to verify the usability and significance of this method for the research of lipids and other valuable long chain hydrocarbons in both wild-type and metabolically engineered production hosts.
LP 23	[62]	The blubber adipocyte index: A nondestructive biomarker of adiposity in humpback whales (*Megaptera novaeangliae*)	To validate sensitivity of adipocytes metrics and the rapid, nonlethal, and inexpensive nature of the methodology and the inclusion of AI in long-term monitoring of humpback whale population health, and further raise the potential for broader wildlife applications.
LP 24	[63]	Fatty acids in common minke whale (*Balaenoptera acutorostrata*) blubber reflect the feeding area and food selection, but also high endogenous metabolism	To analyze ecological abundance or biomass data in comparison with FA data.
LP25	[64]	Isovaleric acid accumulation in odontocete melon during development	To apply strong selective advantage for the maintenance of intimate, long-term calf/guardian relationships in these species.
LP 26	[65]	Function and evolution of specialized endogenous lipids in toothed whales	To understand the unique lipid synthesis pathways that allow deposition of an unusual suite of endogenous lipids [including wax esters and branched-chain fatty acids (BCFA)] into the adipose tissues of toothed whales. To elucidate specific molecular mechanisms controlling the synthesis and deposition of wax esters and branched-chain fatty acids, as well as their spatial deposition within tissues and within adipocytes. To find potential

			metabolomics and genomics applications for further understanding of lipid metabolism. To use matrix-assisted laser desorption/ionization imaging mass-spectrometry to understand the evolution and molecular physiology of these unusual lipids.
LP 27	[66]	Recent advances in expanding the coverage of the lipidome	To actively populate existing public databases for the community and combinations of sample extraction and separation procedures.
LP 28	[67]	Phylogenomic reconstruction of archaeal fatty acid metabolism	To explore biofuel production and other industrial applications by archaeal enzymes involved with fatty acid metabolism. To experimentally confirm the expected pathway of fatty acid production.
LP 29	[68]	The Role of Lipid Biomarkers in Major Depression	To sub-categorize within major depressive disorder such that targeting omega-3 PUFA-sensitive depression
LP 30	[69]	A review of lipidomics technologies applicable to sphingolipidomics and their relevant applications	To develop technologies that enable us to study cellular sphingolipid molecular species temporally, spatially, and dynamically.
LP 31	[70]	Identifying key membrane protein lipid interactions using mass spectrometry	To compare the lipid profile of the host organism with that of the native organism.
LP 32	[71]	Stratification of lipids, fatty acids and organochlorine contaminants in blubber of white whales and killer whales	To apply additional biomarkers and analytical methods specifically for biopsy samples, so each small sample can provide a large array of data about the animal's life history, body condition and health.
LP 33	[72]	The BUME method: a new rapid and simple chloroform-free method for total lipid extraction of animal tissue	The new BUME methods for biofluids and tissue samples presented here are superior to the old, laborious and toxic techniques.
LP 34	[73]	"Bligh and Dyer" and Folch Methods for Solid–Liquid–Liquid Extraction of Lipids from Microorganisms. Comprehension of Solvatation Mechanisms and towards Substitution with Alternative Solvents	To consider use of greener solvents and bio-sourced solvents as alternatives.
LP 35	[74]	Metabolism of ingested lipids by North Atlantic right whales	To further refine models of habitat quality (in terms of the nutritional value of the prey base) for right whales, and in turn aid the creation of management strategies aimed at protecting critical habitat for these animals.
LP 36	[75]	Fat body, fat pad and adipose tissues in invertebrates and vertebrates: the nexus	To identify the fat droplet proteomes and sub-proteomes and their defects.
LP37	[76]	Topographical Distribution of Lipids Inside the Mandibular Fat Bodies of Odontocetes: Remarkable Complexity and Consistency	To identify specific physical implications for sound transmission and reception, based on the differences in branched-chain acids in the jaw fats of toothed whales. To confirm the uniformity of this lipid distribution pattern in additional species and specimens and in complete ontogenetic series; determination of physical properties (melting point, phase-change properties) of key lipid constituents; empirical data on acoustic velocity through branched-chain lipids and WE; a better understanding of the metabolism of these unusual

			endogenous lipids; and studies on the development of echolocation and sound perception.
IN1	[11]	An integrative systems genetic analysis of mammalian lipid metabolism	To apply trans-omic analyses using these datasets facilitated the identification and validation of PSMD9 as a previously unknown lipid regulatory protein. To provide opportunities for the discovery of therapeutic agents and biomarkers in the setting of hepatic lipotoxicity.
IN2	[10]	Identification of the Wax Ester Synthase/Acyl-Coenzyme A:Diacylglycerol Acyltransferase WSD1 Required for Stem Wax Ester Biosynthesis in Arabidopsis[1,2][W][OA]	To study more on *WSD1* to restore TAG biosynthesis in this yeast mutant.
IN3	[77]	Combined genetic and transcriptome analysis of patients with SLE: distinct, targetable signatures for susceptibility and severity	To characterize the 'genomic architecture' of SLE provides additional clues to the understanding of the systemic nature of the disease, its marked heterogeneity and novel targets of therapy and biomarkers for diagnosis/monitoring.
IN4	[78]	Genome, transcriptome and proteome: the rise of omics data and their integration in biomedical sciences	To find knowledge outside of their field of expertise and fosters a leap from the reductionist to the global-integrative analytical approach in research. To use interdisciplinary data integration strategies to support a better understanding of biological systems and eventually the development of successful precision medicine.
IN5	[79]	Rise of Deep Learning for Genomic, Proteomic, and Metabolomic Data Integration in Precision Medicine	To effectively model and integrate almost any type of omics and other data given enough context and scale. To use custom analyses and domain expert interpretation for medical data
IN6	[80]	Integrating multi-omics and regular analyses identifies the molecular responses of zebrafish brains to graphene oxide: Perspectives in environmental criteria	To combine multi-omics and regular analyses provides insights into the actual and highly influential mechanisms underlying nanotoxicity.
IN7	[81]	Is the Nutritional Value of Fish Fillet Related to Fish Maturation or Fish Age? Integrated Analysis of Transcriptomics and Metabolomics in Blunt Snout Bream (*Megalobrama amblycephala*)	To identify genes, including fumarate hydratase (c992_g1) and GNPAT (52107_g1), as promising regulatory candidates in glycerphospholipid metabolism, as well as ACOT1/2/4 (22947_g2) in steroid biosynthesis and their interaction.
IN8	[82]	Integrative Omics Reveals Metabolic and Transcriptomic Alteration of Nonalcoholic Fatty Liver Disease in Catalase Knockout Mice	To study new pathways between transcriptomics and metabolomics.
IN9	[83]	A Multi-Omics Analysis Pipeline for the Metabolic Pathway Reconstruction in the Orphan Species Quercus ilex	To carry out data validation and integration at the different molecular levels. To obtain an unbiased molecular interpretation of the plant biology. To use alternative and complementary strategies such as LC-MS to visualize pathways.
IN10	[84]	Tick Gené's organ engagement in lipid	To study egg viability and development in the

	metabolism revealed by a combined transcriptomic and proteomic approach	environment, thus identifying potential targets for new tick control methods.

Note; No. indicates reference papers in different omics technologies. LN; lipidomics, TP; Transcriptomics, GN; genomics, PR; proteomics and IN; integrated multi-omics.

References

1. Jha, P.; Mcdevitt, M.T.; Halilbasic, E.; Williams, E.G.; Pedro, M.; Gariani, K.; Sleiman, M.B.; Gupta, R.; Ulbrich, A.; Coon, J.J.; et al. Genetic Regulation of Plasma Lipid Species and Their Association with Metabolic Phenotypes. **2019**, *6*, 709–721, doi:10.1016/j.cels.2018.05.009.Genetic.
2. Lapsys, N.M.; Kriketos, A.D.; Lim-Fraser, M.; Furler, S.M.; Chisholm, D.J.; Lowy, A.; Poynten, A.M.; Cooney, G.J. Expression of Genes Involved in Lipid Metabolism Correlate with Peroxisome Proliferator-Activated Receptor γ Expression in Human Skeletal Muscle 1 . *J. Clin. Endocrinol. Metab.* **2014**, *85*, 4293–4297, doi:10.1210/jcem.85.11.6973.
3. Kuivenhoven, J.A.; Hegele, R.A. Mining the genome for lipid genes. *Biochim. Biophys. Acta - Mol. Basis Dis.* **2014**, *1842*, 1993–2009, doi:10.1016/j.bbadis.2014.04.028.
4. Sharma, V.; Hecker, N.; Roscito, J.G.; Foerster, L.; Langer, B.E.; Hiller, M. A genomics approach reveals insights into the importance of gene losses for mammalian adaptations. *Nat. Commun.* **2018**, *9*, doi:10.1038/s41467-018-03667-1.
5. Tollis, M.; Robbins, J.; Webb, A.E.; Kuderna, L.F.K.; Caulin, A.F.; Garcia, J.D.; Bèrubè, M.; Pourmand, N.; Marques-Bonet, T.; O'Connell, M.J.; et al. Return to the sea, get huge, beat cancer: an analysis of cetacean genomes including an assembly for the humpback whale (Megaptera novaeangliae). *Mol. Biol. Evol.* **2019**, doi:10.1093/molbev/msz099.
6. Yang, X.; Lee, W.-P.; Ye, K.; Lee, C. One reference genome is not enough. *Genome Biol.* **2019**, *20*, 104, doi:10.1186/s13059-019-1717-0.
7. Himes, K.P.; Young, A.; Koppes, E.; Stolz, D.; Barak, Y.; Sadovsky, Y.; Chaillet, J.R. Loss of inherited genomic imprints in mice leads to severe disruption in placental lipid metabolism. *Placenta* **2015**, *36*, 389–396, doi:10.1016/j.placenta.2015.01.012.
8. Ball, H.C.; Londraville, R.L.; Prokop, J.W.; George, J.C.; Suydam, R.S.; Vinyard, C.; Thewissen, J.G.M.; Duff, R.J. Beyond thermoregulation: metabolic function of cetacean blubber in migrating bowhead and beluga whales. *J. Comp. Physiol. B Biochem. Syst. Environ. Physiol.* **2017**, *187*, 235–252, doi:10.1007/s00360-016-1029-6.
9. Endo, Y.; Kamei, K.-I.; Inoue-Murayama, M. Genetic Signatures of Lipid Metabolism Evolution in Cetacea. **2018**, doi:10.1101/250597.
10. Li, F.; Wu, X.; Lam, P.; Bird, D.; Zheng, H.; Samuels, L.; Jetter, R.; Kunst, L. Identification of the wax ester synthase/acyl-coenzyme a:diacylglycerol acyltransferase WSD1 required for stem wax ester biosynthesis in Arabidopsis. *Plant Physiol.* **2008**, *148*, 97–107, doi:10.1104/pp.108.123471.
11. Parker, B.L.; Calkin, A.C.; Seldin, M.M.; Keating, M.F.; Tarling, E.J.; Yang, P.; Moody, S.C.; Liu, Y.; Zerenturk, E.J.; Needham, E.J.; et al. An integrative systems genetic analysis of mammalian lipid metabolism. *Nature* **2019**, *567*, 187–193, doi:10.1038/s41586-019-0984-y.
12. Wang, Z.; Chen, Z.; Xu, S.; Ren, W.; Zhou, K.; Yang, G. "Obesity" is healthy for cetaceans? Evidence from pervasive positive selection in genes related to triacylglycerol metabolism. *Sci. Rep.* **2015**, *5*, 1–12, doi:10.1038/srep14187.
13. Cases, S.; Smith, S.J.; Zheng, Y.-W.; Myers, H.M.; Lear, S.R.; Sande, E.; Novak, S.; Collins, C.; Welch, C.B.; Lusis, A.J.; et al. Identification of a gene encoding an acyl CoA:diacylglycerol acyltransferase, a key enzyme in triacylglycerol synthesis. *Proc. Natl. Acad. Sci.* **1998**, *95*, 13018–13023, doi:10.1073/pnas.95.22.13018.
14. Podechard, N.; Ducheix, S.; Polizzi, A.; Lasserre, F.; Montagner, A.; Legagneux, V.; Fouché, E.; Saez, F.; Lobaccaro, J.M.; Lakhal, L.; et al. Dual extraction of mRNA and lipids from a single biological sample. *Sci. Rep.* **2018**, *8*, 1–11, doi:10.1038/s41598-018-25332-9.
15. Zhang, D.G.; Cheng, J.; Tai, Z.P.; Luo, Z. Identification of five genes in endoplasmic reticulum (ER) stress–apoptosis pathways

in yellow catfish Pelteobagrus fulvidraco and their transcriptional responses to dietary lipid levels. *Fish Physiol. Biochem.* **2019**, doi:10.1007/s10695-019-00627-4.

16. Khudyakov, J.I.; Champagne, C.D.; Meneghetti, L.M.; Crocker, D.E. Blubber transcriptome response to acute stress axis activation involves transient changes in adipogenesis and lipolysis in a fasting-adapted marine mammal. *Sci. Rep.* **2017**, *7*, 1–12, doi:10.1038/srep42110.
17. Corominas, J.; Ramayo-Caldas, Y.; Puig-Oliveras, A.; Estellé, J.; Castelló, A.; Alves, E.; Pena, R.N.; Ballester, M.; Folch, J.M. Analysis of porcine adipose tissue transcriptome reveals differences in de novo fatty acid synthesis in pigs with divergent muscle fatty acid composition. *BMC Genomics* **2013**, *14*, 843, doi:10.1186/1471-2164-14-843.
18. Ierardi, J.L.; Mancia, A.; McMillan, J.; Lundqvist, M.L.; Romano, T.A.; Wise, J.P.; Warr, G.W.; Chapman, R.W. Sampling the skin transcriptome of the North Atlantic right whale. *Comp. Biochem. Physiol. - Part D Genomics Proteomics* **2009**, *4*, 154–158, doi:10.1016/j.cbd.2009.01.004.
19. Morey, J.S.; Burek Huntington, K.A.; Campbell, M.; Clauss, T.M.; Goertz, C.E.; Hobbs, R.C.; Lunardi, D.; Moors, A.J.; Neely, M.G.; Schwacke, L.H.; et al. De novo transcriptome assembly and RNA-Seq expression analysis in blood from beluga whales of Bristol Bay, AK. *Mar. Genomics* **2017**, *35*, doi:10.1016/j.margen.2017.08.001.
20. Cui, Y.; Zheng, Y.; Liu, X.; Yan, L.; Fan, X.; Yong, J.; Hu, Y.; Dong, J.; Li, Q.; Wu, X.; et al. Single-Cell Transcriptome Analysis Maps the Developmental Track of the Human Heart. *Cell Rep.* **2019**, *26*, 1934-1950.e5, doi:10.1016/j.celrep.2019.01.079.
21. Ma, S.; Zhou, X.; Gerashchenko, M. V; Lee, S.G.; George, J.C.; Bickham, J.W.; Gladyshev, V.N.; Suydam, R.; Seim, I. The transcriptome of the bowhead whale *Balaena mysticetus* reveals adaptations of the longest - lived mammal. *Aging (Albany. NY).* **2014**, *6*, 879–899, doi:10.18632/aging.100699.
22. Yao, C.; Pang, D.; Lu, C.; Xu, A.; Huang, P.; Ouyang, H.; Yu, H. Data Mining and Validation of AMPK Pathway as a Novel Candidate Role Affecting Intramuscular Fat Content in Pigs. *Animals* **2019**, *9*, 137, doi:10.3390/ani9040137.
23. Cui, J.X.; Zeng, Q.F.; Chen, W.; Zhang, H.; Zeng, Y.Q. Analysis and preliminary validation of the molecular mechanism of fat deposition in fatty and lean pigs by high-throughput sequencing. *Mamm. Genome* **2019**, *30*, 71–80, doi:10.1007/s00335-019-09795-3.
24. Cai, H.; Li, M.; Sun, X.; Plath, M.; Li, C.; Lan, X.; Lei, C.; Huang, Y.; Bai, Y.; Qi, X.; et al. Global transcriptome analysis during adipogenic differentiation and involvement of transthyretin gene in adipogenesis in cattle. *Front. Genet.* **2018**, *9*, 1–13, doi:10.3389/fgene.2018.00463.
25. Luecken, M.D.; Theis, F.J. Current best practices in single-cell RNA-seq analysis: a tutorial. *Mol. Syst. Biol.* **2019**, *15*, e8746, doi:10.15252/msb.20188746.
26. Vigors, S.; O'Doherty, J. V.; Bryan, K.; Sweeney, T. A comparative analysis of the transcriptome profiles of liver and muscle tissue in pigs divergent for feed efficiency. *BMC Genomics* **2019**, *20*, 461, doi:10.1186/s12864-019-5740-z.
27. McGinnis, C.S.; Patterson, D.M.; Winkler, J.; Conrad, D.N.; Hein, M.Y.; Srivastava, V.; Hu, J.L.; Murrow, L.M.; Weissman, J.S.; Werb, Z.; et al. MULTI-seq: sample multiplexing for single-cell RNA sequencing using lipid-tagged indices. *Nat. Methods* **2019**, *16*, 619–626, doi:10.1038/s41592-019-0433-8.
28. Han, Y.; Gao, S.; Muegge, K.; Zhang, W.; Zhou, B. Advanced applications of RNA sequencing and challenges. *Bioinform. Biol. Insights* **2015**, *9*, 29–46, doi:10.4137/BBI.S28991.
29. Viguerie, N.; Montastier, E.; Maoret, J.J.; Roussel, B.; Combes, M.; Valle, C.; Villa-Vialaneix, N.; Iacovoni, J.S.; Martinez, J.A.; Holst, C.; et al. Determinants of Human Adipose Tissue Gene Expression: Impact of Diet, Sex, Metabolic Status, and Cis Genetic Regulation. *PLoS Genet.* **2012**, *8*, doi:10.1371/journal.pgen.1002959.
30. Resnyk, C.W.; Chen, C.; Huang, H.; Wu, C.H.; Simon, J.; Le Bihan-Duval, E.; Duclos, M.J.; Cogburn, L.A. RNA-seq analysis of abdominal fat in genetically fat and lean chickens highlights a divergence in expression of genes controlling adiposity, hemostasis, and lipid metabolism. *PLoS One* **2015**, *10*, 1–41, doi:10.1371/journal.pone.0139549.

31. Cotter, D. LMPD: LIPID MAPS proteome database. *Nucleic Acids Res.* **2005**, *34*, D507–D510, doi:10.1093/nar/gkj122.
32. Swan, A.L.; Mobasheri, A.; Allaway, D.; Liddell, S.; Bacardit, J. Application of Machine Learning to Proteomics Data: Classification and Biomarker Identification in Postgenomics Biology. *Omi. A J. Integr. Biol.* **2013**, *17*, 595–610, doi:10.1089/omi.2013.0017.
33. Kershaw, J.L.; Botting, C.H.; Brownlow, A.; Hall, A.J. Not just fat: Investigating the proteome of cetacean blubber tissue. *Conserv. Physiol.* **2018**, *6*, 1–15, doi:10.1093/conphys/coy003.
34. Yang, L.; Ding, Y.; Chen, Y.; Zhang, S.; Huo, C.; Wang, Y.; Yu, J.; Zhang, P.; Na, H.; Zhang, H.; et al. The proteomics of lipid droplets: structure, dynamics, and functions of the organelle conserved from bacteria to humans. *J. Lipid Res.* **2012**, *53*, 1245–1253, doi:10.1194/jlr.r024117.
35. Wang, X.; Wei, H.; Mao, X.; Liu, J. Proteomics Analysis of Lipid Droplets from the Oleaginous Alga Chromochloris zofingiensis Reveals Novel Proteins for Lipid Metabolism. *Genomics. Proteomics Bioinformatics* **2019**, *17*, 260–272, doi:10.1016/j.gpb.2019.01.003.
36. Chen, J.; Huang, J.; Deng, J.; Ma, H.; Zou, S. Use of comparative proteomics to identify the effects of creatine pyruvate on lipid and protein metabolism in broiler chickens. *Vet. J.* **2012**, *193*, 514–521, doi:10.1016/j.tvjl.2012.01.034.
37. Choi, Y.E.; Hwang, H.; Kim, H.S.; Ahn, J.W.; Jeong, W.J.; Yang, J.W. Comparative proteomics using lipid over-producing or less-producing mutants unravels lipid metabolisms in Chlamydomonas reinhardtii. *Bioresour. Technol.* **2013**, doi:10.1016/j.biortech.2013.03.142.
38. Zhao, L.; Du, M.; Gao, J.; Zhan, B.; Mao, X. Label-free quantitative proteomic analysis of milk fat globule membrane proteins of yak and cow and identification of proteins associated with glucose and lipid metabolism. *Food Chem.* **2019**, *275*, 59–68, doi:10.1016/j.foodchem.2018.09.044.
39. Wu, Y.; Tang, J.; Zhou, C.; Zhao, L.; Chen, J.; Zeng, L.; Rao, C.; Shi, H.; Liao, L.; Liang, Z.; et al. Quantitative proteomics analysis of the liver reveals immune regulation and lipid metabolism dysregulation in a mouse model of depression. *Behav. Brain Res.* **2016**, *311*, 330–339, doi:10.1016/j.bbr.2016.05.057.
40. Yadetie, F.; Oveland, E.; Døskeland, A.; Berven, F.; Goksøyr, A.; Karlsen, O.A. Quantitative proteomics analysis reveals perturbation of lipid metabolic pathways in the liver of Atlantic cod (Gadus morhua) treated with PCB 153. *Aquat. Toxicol.* **2017**, doi:10.1016/j.aquatox.2017.01.014.
41. Ashrafi, K. Obesity and the regulation of fat metabolism. *WormBook* **2007**, 1–20, doi:10.1895/wormbook.1.130.1.
42. Nelson, D.W.; Eric Yen, C.-L. Triacylglycerol synthesis and energy metabolism: a gut reaction? *Clin. Lipidol.* **2009**, *4*, 683–686, doi:10.2217/clp.09.67.
43. Baker, P.R.S.; Armando, A.M.; Campbell, J.L.; Quehenberger, O.; Dennis, E.A. Three-dimensional enhanced lipidomics analysis combining UPLC, differential ion mobility spectrometry, and mass spectrometric separation strategies. *J. Lipid Res.* **2014**, *55*, 2432–2442, doi:10.1194/jlr.d051581.
44. Yamato, M.; Koopman, H.; Niemeyer, M.; Ketten, D. Characterization of lipids in adipose depots associated with minke and fin whale ears: Comparison with "acoustic fats" of toothed whales. *Mar. Mammal Sci.* **2014**, *30*, 1549–1563, doi:10.1111/mms.12120.
45. Jung, J.L.; Simon, G.; Alfonsi, E.; Thoraval, D.; Kervarec, N.; Ben Salem, D.; Hassani, S.; Domergue, F. Qualitative and quantitative study of the highly specialized lipid tissues of cetaceans using HR-MAS NMR and classical GC. *PLoS One* **2017**, *12*, 1–23, doi:10.1371/journal.pone.0180597.
46. Ulmer, C.Z.; Jones, C.M.; Yost, R.A.; Garrett, T.J.; Bowden, J.A. Optimization of Folch, Bligh-Dyer, and Matyash sample-to-extraction solvent ratios for human plasma-based lipidomics studies. *Anal. Chim. Acta* **2018**, *1037*, 351–357, doi:10.1016/j.aca.2018.08.004.
47. Röttig, A.; Zurek, P.J.; Steinbüchel, A. Assessment of bacterial acyltransferases for an efficient lipid production in

metabolically engineered strains of E. coli. *Metab. Eng.* **2015**, *32*, 195–206, doi:10.1016/j.ymben.2015.09.016.
48. Tyagi, M.G.; Varshney, P.; Sagar, K.D.; Nirmala, J. Recent Analytical Trends in Lipidomics®; Techniques and Applications in Clinical Medicine. *J. Pharm. Biol. Sci.* **2016**, *11*, 88–92, doi:10.9790/3008-1106068892.
49. Barnes, S.; Benton, H.P.; Casazza, K.; Cooper, S.J.; Cui, X.; Du, X.; Engler, J.; Kabarowski, J.H.; Li, S.; Pathmasiri, W.; et al. Training in metabolomics research. I. Designing the experiment, collecting and extracting samples and generating metabolomics data. *J. Mass Spectrom.* **2016**, 461–475, doi:10.1002/jms.3782.
50. Boggs, A.S.P.; Schock, T.B.; Schwacke, L.H.; Galligan, T.M.; Morey, J.S.; McFee, W.E.; Kucklick, J.R. Rapid and reliable steroid hormone profiling in Tursiops truncatus blubber using liquid chromatography tandem mass spectrometry (LC-MS/MS). *Anal. Bioanal. Chem.* **2017**, *409*, 5019–5029, doi:10.1007/s00216-017-0446-z.
51. Li, L.; Han, J.; Wang, Z.; Liu, J.; Wei, J.; Xiong, S.; Zhao, Z. Mass spectrometry methodology in lipid analysis. *Int. J. Mol. Sci.* **2014**, *15*, 10492–10507, doi:10.3390/ijms150610492.
52. Shevchenko, A.; Simons, K. diversity. **2010**.
53. Hashimoto, O.; Ohtsuki, H.; Kakizaki, T.; Amou, K.; Sato, R.; Doi, S.; Kobayashi, S.; Matsuda, A.; Sugiyama, M.; Funaba, M.; et al. Brown adipose tissue in cetacean blubber. *PLoS One* **2015**, *10*, 1–14, doi:10.1371/journal.pone.0116734.
54. Bozek, K.; Khrameeva, E.E.; Reznick, J.; Omerbašić, D.; Bennett, N.C.; Lewin, G.R.; Azpurua, J.; Gorbunova, V.; Seluanov, A.; Regnard, P.; et al. Lipidome determinants of maximal lifespan in mammals. *Sci. Rep.* **2017**, *7*, 1–10, doi:10.1038/s41598-017-00037-7.
55. Chen, H.K.; Wang, L.H.; Chen, W.N.U.; Mayfield, A.B.; Levy, O.; Lin, C.S.; Chen, C.S. Coral lipid bodies as the relay center interconnecting diel-dependent lipidomic changes in different cellular compartments. *Sci. Rep.* **2017**, *7*, 1–13, doi:10.1038/s41598-017-02722-z.
56. Pham, T.H.; Zaeem, M.; Fillier, T.A.; Nadeem, M.; Vidal, N.P.; Manful, C.; Cheema, S.; Cheema, M.; Thomas, R.H. Targeting Modified Lipids during Routine Lipidomics Analysis using HILIC and C30 Reverse Phase Liquid Chromatography coupled to Mass Spectrometry. *Sci. Rep.* **2019**, *9*, 1–15, doi:10.1038/s41598-019-41556-9.
57. Sadowski, T.; Klose, C.; Gerl, M.J.; Wójcik-Maciejewicz, A.; Herzog, R.; Simons, K.; Reich, A.; Surma, M.A. Large-scale human skin lipidomics by quantitative, high-throughput shotgun mass spectrometry. *Sci. Rep.* **2017**, *7*, 1–11, doi:10.1038/srep43761.
58. Duggan, Z.P.Z.; Koopman, H.N.; Budge, S.M. Distribution and development of the highly specialized lipids in the sound reception systems of dolphins. *J. Comp. Physiol. B Biochem. Syst. Environ. Physiol.* **2009**, *179*, 783–798, doi:10.1007/s00360-009-0360-6.
59. Coleman, R.A.; Mashek, D.G. Mammalian triacylglycerol metabolism: Synthesis, lipolysis, and signaling. *Chem. Rev.* 2011, *111*, 6359–6386.
60. Lange, M.; Ni, Z.; Criscuolo, A.; Fedorova, M. Liquid Chromatography Techniques in Lipidomics Research. *Chromatographia* **2019**, *82*, 77–100, doi:10.1007/s10337-018-3656-4.
61. Santala, S.; Efimova, E.; Karp, M.; Santala, V. Real-Time monitoring of intracellular wax ester metabolism. *Microb. Cell Fact.* **2011**, *10*, 75, doi:10.1186/1475-2859-10-75.
62. Castrillon, J.; Huston, W.; Bengtson Nash, S. The blubber adipocyte index: A nondestructive biomarker of adiposity in humpback whales (Megaptera novaeangliae). *Ecol. Evol.* **2017**, *7*, 5131–5139, doi:10.1002/ece3.2913.
63. Meier, S.; Falk-Petersen, S.; Aage Gade-Sørensen, L.; Greenacre, M.; Haug, T.; Lindstrøm, U. Fatty acids in common minke whale (Balaenoptera acutorostrata) blubber reflect the feeding area and food selection, but also high endogenous metabolism. *Mar. Biol. Res.* **2016**, *12*, 221–238, doi:10.1080/17451000.2015.1118513.
64. Gardner, S.C.; Varanasi, U. Isovaleric acid accumulation in odontocete melon during development. *Naturwissenschaften* **2003**, *90*, 528–531, doi:10.1007/s00114-003-0472-x.
65. Koopman, H.N. Function and evolution of specialized endogenous lipids in toothed whales. *J. Exp. Biol.* **2018**, *221*, jeb161471,

doi:10.1242/jeb.161471.
66. Tumanov, S.; Kamphorst, J.J. Recent advances in expanding the coverage of the lipidome. *Curr. Opin. Biotechnol.* **2017**, *43*, 127–133, doi:10.1016/j.copbio.2016.11.008.
67. Dibrova, D. V.; Galperin, M.Y.; Mulkidjanian, A.Y. Phylogenomic reconstruction of archaeal fatty acid metabolism. *Environ. Microbiol.* **2014**, *16*, 907–918, doi:10.1111/1462-2920.12359.
68. Parekh, A.; Smeeth, D.; Milner, Y.; Thuret, S. The Role of Lipid Biomarkers in Major Depression. *Healthcare* **2017**, *5*, 5, doi:10.3390/healthcare5010005.
69. Han, X.; Jiang, X. A review of lipidomic technologies applicable to sphingolipidomics and their relevant applications. *Eur. J. Lipid Sci. Technol.* 2009, *111*, 39–52.
70. Gupta, K.; Li, J.; Liko, I.; Gault, J.; Bechara, C.; Wu, D.; Hopper, J.T.S.; Giles, K.; Benesch, J.L.P.; Robinson, C. V. Identifying key membrane protein lipid interactions using mass spectrometry. *Nat. Protoc.* **2018**, *13*, 1106–1120, doi:10.1038/nprot.2018.014.
71. Krahn, M.M.; Herman, D.P.; Ylitalo, G.M.; Sloan, C.A.; Burrows, D.G.; Hobbs, R.C.; Mahoney, B.A.; Yanagida, G.K.; Calambokidis, J.; Moore, S.E. Stratification of lipids, fatty acids and organochlorine contaminants in blubber of white whales and killer whales. *J. Cetacean Res. Manag.* **2004**, *6*, 175–189.
72. Löfgren, L.; Forsberg, G.B.; Ståhlman, M. The BUME method: A new rapid and simple chloroform-free method for total lipid extraction of animal tissue. *Sci. Rep.* **2016**, *6*, 1–11, doi:10.1038/srep27688.
73. Breil, C.; Abert Vian, M.; Zemb, T.; Kunz, W.; Chemat, F. "Bligh and Dyer" and Folch methods for solid–liquid–liquid extraction of lipids from microorganisms. Comprehension of solvatation mechanisms and towards substitution with alternative solvents. *Int. J. Mol. Sci.* **2017**, *18*, 1–21, doi:10.3390/ijms18040708.
74. Swaim, Z.T.; Westgate, A.J.; Koopman, H.N.; Rolland, R.M.; Kraus, S.D. Metabolism of ingested lipids by North Atlantic right whales. *Endanger. Species Res.* **2009**, *6*, 259–271, doi:10.3354/esr00163.
75. Azeez, O.I.; Meintjes, R.; Chamunorwa, J.P. Fat body, fat pad and adipose tissues in invertebrates and vertebrates: The nexus. *Lipids Health Dis.* **2014**, *13*, 1–13, doi:10.1186/1476-511X-13-71.
76. Koopman, H.N.; Budge, S.M.; Ketten, D.R.; Iverson, S.J. Topographical distribution of lipids inside the mandibular fat bodies of odontocetes: Remarkable complexity and consistency. *IEEE J. Ocean. Eng.* **2006**, *31*, 95–106, doi:10.1109/JOE.2006.872205.
77. Panousis, N.I.; Bertsias, G.K.; Ongen, H.; Gergianaki, I.; Tektonidou, M.G.; Trachana, M.; Romano-Palumbo, L.; Bielser, D.; Howald, C.; Pamfil, C.; et al. Combined genetic and transcriptome analysis of patients with SLE: distinct, targetable signatures for susceptibility and severity. *Ann. Rheum. Dis.* **2019**, *78*, 1079–1089, doi:10.1136/annrheumdis-2018-214379.
78. Manzoni, C.; Kia, D.A.; Vandrovcova, J.; Hardy, J.; Wood, N.W.; Lewis, P.A.; Ferrari, R. Genome, transcriptome and proteome: The rise of omics data and their integration in biomedical sciences. *Brief. Bioinform.* **2018**, *19*, 286–302, doi:10.1093/BIB/BBW114.
79. Grapov, D.; Fahrmann, J.; Wanichthanarak, K.; Khoomrung, S. Rise of Deep Learning for Genomic, Proteomic, and Metabolomic Data Integration in Precision Medicine. *Omi. A J. Integr. Biol.* **2018**, *22*, 630–636, doi:10.1089/omi.2018.0097.
80. Sun, J.; Zhou, Q.; Hu, X. Integrating multi-omics and regular analyses identifies the molecular responses of zebrafish brains to graphene oxide: Perspectives in environmental criteria. *Ecotoxicol. Environ. Saf.* **2019**, *180*, 269–279, doi:10.1016/j.ecoenv.2019.05.011.
81. Guan, N.N.; Zhou, Q.; Lan, T.; Zhou, L.F.; Zhao, B.W.; Wang, W.M.; Gao, Z.X. Is the Nutritional Value of Fish Fillet Related to Fish Maturation or Fish Age? Integrated Analysis of Transcriptomics and Metabolomics in Blunt Snout Bream (Megalobrama amblycephala). *Cell. Physiol. Biochem.* **2018**, *49*, 17–39, doi:10.1159/000492837.
82. Na, J.; Choi, S.A.; Khan, A.; Huh, J.Y.; Piao, L.; Hwang, I.; Ha, H.; Park, Y.H. Integrative omics reveals metabolic and transcriptomic alteration of nonalcoholic fatty liver disease in catalase knockout mice. *Biomol. Ther.* **2019**, *27*, 134–144, doi:10.4062/biomolther.2018.175.
83. López-Hidalgo, C.; Guerrero-Sánchez, V.M.; Gómez-Gálvez, I.; Sánchez-Lucas, R.; Castillejo-Sánchez, M.A.;

Maldonado-Alconada, A.M.; Valledor, L.; Jorrín-Novo, J. V. A multi-omics analysis pipeline for the metabolic pathway reconstruction in the orphan species quercus ilex. *Front. Plant Sci.* **2018**, *9*, 1–16, doi:10.3389/fpls.2018.00935.

84. Xavier, M.A.; Tirloni, L.; Pinto, A.F.M.; Diedrich, J.K.; Yates, J.R.; Gonzales, S.; Farber, M.; da Silva Vaz, I.; Termignoni, C. Tick Gené's organ engagement in lipid metabolism revealed by a combined transcriptomic and proteomic approach. *Ticks Tick. Borne. Dis.* **2019**, *10*, 787–797, doi:10.1016/j.ttbdis.2019.03.013.
